# Superresolution structured illumination microscopy reconstruction algorithms: a review

**DOI:** 10.1038/s41377-023-01204-4

**Published:** 2023-07-12

**Authors:** Xin Chen, Suyi Zhong, Yiwei Hou, Ruijie Cao, Wenyi Wang, Dong Li, Qionghai Dai, Donghyun Kim, Peng Xi

**Affiliations:** 1 Department of Biomedical Engineering, College of Future Technology, Peking University, Beijing, 100871 China; 2 National Biomedical Imaging Center, Peking University, Beijing, 100871 China; 3 National Laboratory of Biomacromolecules, CAS Center for Excellence in Biomacromolecules, Institute of Biophysics, Chinese Academy of Sciences, Beijing, China; 4 Department of Automation, Tsinghua University, Beijing, China; 5 Institute for Brain and Cognitive Sciences, Tsinghua University, Beijing, China; 6 Beijing Key Laboratory of Multidimension & Multiscale Computational Photography, Tsinghua University, Beijing, China; 7 Beijing Laboratory of Brain and Cognitive Intelligence, Beijing Municipal Education Commission, Beijing, China; 8 School of Electrical and Electronic Engineering, Yonsei University, 50 Yonsei-Ro, Seodaemun-Gu, Seoul, 03722 Korea

**Keywords:** Optical techniques, Optical physics

## Abstract

Structured illumination microscopy (SIM) has become the standard for next-generation wide-field microscopy, offering ultrahigh imaging speed, superresolution, a large field-of-view, and long-term imaging. Over the past decade, SIM hardware and software have flourished, leading to successful applications in various biological questions. However, unlocking the full potential of SIM system hardware requires the development of advanced reconstruction algorithms. Here, we introduce the basic theory of two SIM algorithms, namely, optical sectioning SIM (OS-SIM) and superresolution SIM (SR-SIM), and summarize their implementation modalities. We then provide a brief overview of existing OS-SIM processing algorithms and review the development of SR-SIM reconstruction algorithms, focusing primarily on 2D-SIM, 3D-SIM, and blind-SIM. To showcase the state-of-the-art development of SIM systems and assist users in selecting a commercial SIM system for a specific application, we compare the features of representative off-the-shelf SIM systems. Finally, we provide perspectives on the potential future developments of SIM.

## Introduction

Approximately 400 years ago, Antonie van Leeuwenhoek invented the microscope, ushering in an era of visualizing the biological world with unprecedented detail that surpass the human eye’s resolution^1^. Later, Ernst Abbe theoretically derived the fundamental limit of an optical microscope, stating that the resolution of a microscope is limited by the numerical aperture of the objective and the wavelength of the emitted light^2^. As a result, the lateral- and axial-resolution limits of traditional microscopy are approximately 200 and 500 nm, respectively, restricting its broad applications in research on organelle interactions, cell biology, biomedicine, and related fields. To meet the increasing demand for studying the ultrastructure and interaction of subcellular organelles, various superresolution imaging technologies that surpass the diffraction limit have been developed. These include single-molecule localization microscopy (SMLM)^3–7^, stimulated emission depletion microscopy (STED)^8–10^, and structured illumination microscopy (SIM)^11–14^.

**Fig. 1:**
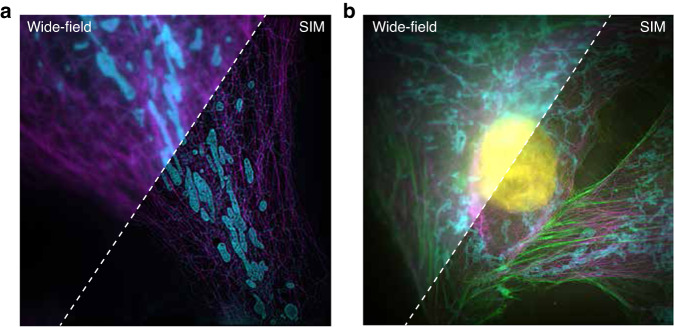
Comparison of a wide-field image and SIM image obtained using the Airy Polar-SIM system^200^. **a** Two-color (561-PK mito RED labeled mitochondria (cyan) and 640-SiR-tubulin kit—labelled tubulin (magenta)) imaging results of homemade sample COS7 cells. Please refer to Appendix 1 for the specific production process. **b** Four-color (DAPI-labelled nuclei, yellow; Alexa 647-labelled tubulin, magenta; Alexa 555-labelled actin, green; Alexa 488-labelled mitochondria, blue) imaging results of fixed cells. The sample was purchased from Standard Imaging Co. Ltd

**Fig. 2:**
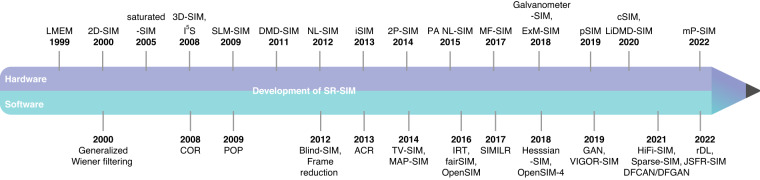
Timeline of landmark work in the SR-SIM field. This timeline highlights some key milestones and advancements in the SR-SIM field, encompassing both hardware and software developments

SIM was originally developed as a depth discrimination method to eliminate out-of-focus contributions from different vertical image planes, a method termed optical sectioning SIM (OS-SIM)^15–19^. Subsequently, superresolution SIM (SR-SIM) was developed, which utilizes a periodic interference pattern with a periodicity near the optical diffraction limit^20^. Due to its ability to cover a variety of wide-field imaging modes (from volumetric imaging to laminar imaging, such as total internal reflection fluorescence (TIRF), highly inclined and laminated optical sheets, or conventional wide-field mode) and its compatibility with conventional fluorescent probes and protocols (shown in Fig. 1), SR-SIM has become the *de facto* standard for live-cell superresolution microscopy. Furthermore, as illustrated in Fig. 2, with the ongoing development of SR-SIM hardware and software, it can offer ultrahigh imaging speeds (>500 frames per second (fps)), superresolution (<100 nm), a large field-of-view (>200 µm), and long-term imaging (>1 h). The superior SR-SIM spatial-temporal bandwidth results outperform other superresolution techniques, particularly for live-cell superresolution imaging.

**Fig. 3:**
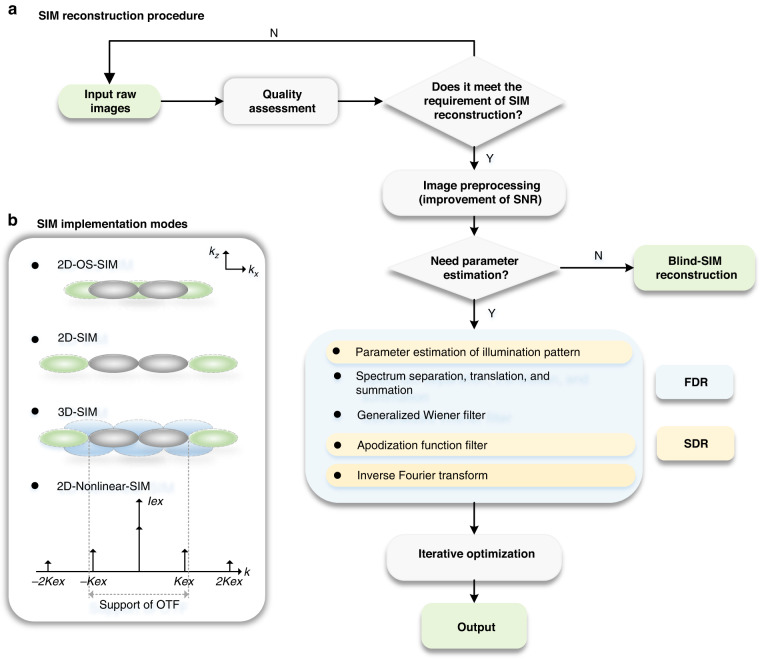
SIM reconstruction procedure and implementation methods. **a** Schematic diagram of the SR-SIM reconstruction process. The substeps in the blue box are used for the Fourier domain reconstruction (FDR) algorithm. In contrast, the spatial domain reconstruction (SDR) algorithm does not involve Fourier transform operations. The ‘iterative optimization’ box represents further optimization of the reconstruction results based on regularization. **b** The first three subpictures represent different SIM implementation modalities, taking linear SIM as an example. The last subpicture is a nonlinear SIM diagram in the Fourier domain. The symbol ‘*Kex*’ represents the frequency vector of the structured illumination pattern *I*_*ex*_

Recently, to further advance the development of SIM technology towards deeper depth, higher resolution, better quality, and faster speed, various methods have been proposed, including improvements in system design and reconstruction algorithms. Figure 3b shows that the implementation modalities for SIM can be summarized in three aspects: enhancing axial resolution (mainly referring to OS-SIM), lateral resolution (2D-SIM, also called two-beam SIM), and both lateral and axial resolution (i.e., the 3D-SIM family, also called three-beam SIM). Both 2D-SIM and 3D-SIM belong to the SR-SIM category. Additionally, SIM can be classified into linear SIM and nonlinear SIM (NL-SIM) based on the harmonic order in the excitation illumination patterns.

The traditional OS-SIM processing algorithm is based on the standard root mean square (RMS), which is a nonlinear reconstruction procedure. In contrast, the original SR-SIM algorithm is based on a linear generalized Wiener filter. The more recently developed SR-SIM algorithms can also provide optical section images by combining notch filtering. As shown in Fig. 3a, we broadly categorize existing SR-SIM reconstruction algorithms into three categories: Fourier domain reconstruction (FDR) algorithm methods, which include the generalized Wiener filter reconstruction method (also known as the direct method) and the regularization-based iterative optimization method, spatial domain reconstruction (SDR) algorithm methods, and blind-SIM reconstruction methods.

Due to its advantages of simplicity and speed, the generalized Wiener filtering reconstruction algorithm is widely used in practice. The regularization-based iterative optimization algorithm proposed later is suitable for various noise models (such as Gaussian or Poisson) and optimization criteria (such as maximum likelihood or maximum entropy), making it more robust to noise. The SDR algorithm has the advantage of faster reconstruction speed compared to the FDR algorithm because it does not involve Fourier transform operations. However, both the FDR and the SDR algorithms rely on sophisticated optical system alignment and precise illumination pattern parameter estimation. To address this problem, the blind-SIM reconstruction algorithm was proposed, which does not require estimating illumination pattern parameters and can improve reconstruction robustness. However, as an iterative solution method, its calculation speed is orders of magnitude slower than the other two methods.

This paper reviews the development of SIM imaging technology in four parts. In the section “Basic SIM theory and its implementation modalities”, we first introduce the SIM principle, including the basic OS-SIM and SR-SIM processing methods. We then review and summarize its implementation modalities, taking linear SIM as an example. Additionally, in the subsection “Nonlinear SIM”, we outline the methods for realizing nonlinear SIM. In the section “Development of SIM reconstruction methods”, we first provide a summary of existing OS-SIM processing algorithms in the subsection “Development of OS-SIM reconstruction methods”. Then, in subsection “Development of 2D-SIM reconstruction methods”, we review the development of 2D-SIM reconstruction methods in detail, mainly focusing on three aspects: (1) the Fourier domain reconstruction (FDR) algorithm, which includes parameter estimation of the structured illumination pattern, generalized Wiener filtering and its improved forms, and the regularization-based iterative optimization algorithm; (2) the spatial domain reconstruction (SDR) algorithm, where we analyse and compare the similarities and differences between SDR and FDR based on the reconstruction procedure; and (3) we summarize existing open-source 2D-SIM image-processing software, and discuss their characteristics and application scope to help readers access the relevant reconstruction tools. In the subsection “Development of 3D-SIM reconstruction methods”, we summarize and compare some typical existing 3D-SIM reconstruction algorithms by processing the same raw 3D-SIM image stacks. Finally, in the subsection “Development of blind-SIM reconstruction methods”, we review the development of blind-SIM reconstruction algorithms. In the section “The combination of SIM with other techniques”, we briefly discuss the combination of SIM with other superresolution technologies. Moreover, we provide a detailed review of the development status of SIM combined with deep-learning techniques. In the section “Summary”, we summarize and compare some representative commercial SIM systems. Finally, we draw conclusions and discuss future perspectives.

## Basic SIM theory and its implementation modalities

### Basic SIM theory

When two periodic patterns with slightly different frequencies *f*_0_ and *f*_1_ are multiplied, a Moiré fringe pattern with a frequency lower than either of the original patterns is produced. What is more interesting is that when one of the patterns is known, the other can be solved algebraically from the Moiré fringe pattern. The resolution of optical microscopy is limited by the wave diffraction nature, which functions as a low-pass filter during the imaging process. As a result, a sample’s fine structure (high-frequency component) cannot pass through the microscope system. However, a Moiré fringe with fine structure information but shifted to low frequency can be obtained, which can be used to resolve the fine structure of the specimen.

SIM technology is based on the phenomenon mentioned above and uses a series of sinusoidal illumination patterns to illuminate an unknown sample. The emitted patterns contain information about the fine details of the unobservable sample structure in a diffraction-limited image. Subsequently, a series of SIM reconstruction procedures can be adopted to determine these fine details of the unknown sample.

#### Basic OS-SIM algorithm

A wide-field fluorescence microscope is typically a partially coherent, low-pass filtering imaging system with an optical transfer function (OTF) support that has a torus-like shape ^21,22^. The system’s lateral and axial resolutions can be described as 2sin(*α*)*n*/*λ* and [1-cos(*α*)]*n*/*λ*, respectively, where *λ* is the wavelength of the emitted light, *α* is the angle between the light beam and optical axis, and *n* is the refractive index of the sample medium. When *α* = 60^0^, the ratio factor between the lateral and axial resolutions is $2\sqrt{3}$, indicating that the axial resolution is approximately three times worse than the lateral resolution. Furthermore, because of the OTF missing cone problem, when acquiring a sequence of 2D images at different focal planes, each image slice contains not only the in-focus information from the corresponding section of the sample but also the out-of-focus blur from all other sections.

To remove the out-of-focus information and obtain the whole structure of a 3D sample, Neil et al.^15,23^ introduced the OS-SIM algorithm. Unlike light-sheet microscopy^24–26^, which uses paired orthogonal optical pathways to confine illumination to a single plane and provides intrinsic optical sectioning images, OS-SIM is an optical computational sectioning imaging technique. By exploiting the phenomenon of attenuating all spatial frequencies except zero with defocus, a single spatial-frequency grid pattern is projected onto the sample, and phase images at different grid pattern positions are captured. A quasiconfocal image can be reconstructed from this set of images using the standard RMS method. If *s*(*r*) denotes the sample, *h*(*r*) represents the point spread function (PSF) of the optical system, and the structured illumination pattern is a cosine function:

1 $${I}_{\theta ,\varphi }(r)=\frac{{I}_{0}}{2}[1+\,\cos (2\pi {k}_{\theta }\cdot r+\varphi )]$$

where *θ* and *φ* represent the angular orientation and spatial phase of the structured illumination pattern, respectively. *I*_0_ represents conventional wide-field illumination. *k*_*θ*_ is the frequency vector of the illumination pattern along the angle *θ*. We assume the illumination pattern modulation depth to be 1. The sample is modulated by the illumination pattern and then convolved with the PSF. The fluorescence signal *D*(*r*) emitted by the sample and detected by the camera can be expressed as:

2 $$D(r)=(s(r)\cdot {I}_{\theta ,\varphi }(r))\otimes h(r)$$

If we substitute Eq. (1) into Eq. (2), we obtain:

3 $$\begin{array}{c}D(r)=\frac{1}{2}[s(r)+s(r)\cdot \,\cos (2\pi {k}_{\theta }\cdot r)\cdot \,\cos (\varphi )-s(r)\cdot \,\sin (2\pi {k}_{\theta }\cdot r)\cdot \,\sin (\varphi )]\otimes h(r)\\ =\frac{1}{2}s(r)\otimes h(r)+\frac{1}{2}\,\cos (\varphi )\cdot \{[s(r)\cdot \,\cos (2\pi {k}_{\theta }\cdot r)]\otimes h(r)\}-\frac{1}{2}\,\sin (\varphi )\cdot \{[s(r)\cdot \,\sin (2\pi {k}_{\theta }\cdot r)]\otimes h(r)\}\\ ={D}_{0}(r)+\,\cos (\varphi )\cdot {D}_{C}(r)-\,\sin (\varphi )\cdot {D}_{S}(r)\end{array}$$

where *D*_0_ represents a conventional wide-field image. ${D}_{C}=s(r)\cdot \,\cos (2\pi {k}_{\theta }\cdot r)$ and ${D}_{S}=s(r)\cdot \,\sin (2\pi {k}_{\theta }\cdot r)$ represent the images resulting from the cosine and sine modulations of the illumination pattern, respectively. The grid pattern can be removed from the specimen image forming ${I}_{P}=\sqrt{{D}_{C}^{2}+{D}_{S}^{2}}$. Typically, three images, namely, *D*_1_, *D*_2_, and *D*_3_, are taken, corresponding to the relative spatial phases $\varphi =0$, $\varphi =2\pi /3$, and $\varphi =4\pi /3$, respectively. An optically sectioned image can be obtained by

4 $${D}_{OS}=\sqrt{{({D}_{1}-{D}_{2})}^{2}+{({D}_{1}-{D}_{3})}^{2}+{({D}_{3}-{D}_{2})}^{2}}$$

The OS-SIM algorithm is based on spatial heterodyning and provides easy system alignment. It yields an optically sectioned image without a grid pattern, but it is not a linear reconstruction procedure. Therefore, the final image *D*_OS_ obtained as the geometric sum of the different images is not shift-invariant. Furthermore, as relatively coarse illumination patterns are used in the OS-SIM system, the purpose of OS-SIM is not to improve the lateral resolution but to add optical sectioning to wide-field microscopy.

#### Basic SR-SIM algorithm

In contrast to OS-SIM, SR-SIM uses a structured illumination pattern with a frequency vector close to the optical diffraction limit. Depending on the number of beams needed to generate the illumination pattern, SR-SIM can be classified into 2D-SIM (two-beam) and 3D-SIM (three-beam). Different illumination patterns are used in SR-SIM to realize resolution enhancement in a single direction (i.e., lateral or axial) or in two directions (both lateral and axial). For example, in 1994, Bailey et al.^16^ designed standing wave illumination microscopy, which utilized two opposing objective lens interferences to generate axially modulated structured illumination patterns. Later, in 1999, Heintzmann et al.^13^ proposed a method called laterally modulated excitation microscopy (LMEM), which processes raw images based on a sample’s information structure in Fourier space. In 2000, Gustafsson^11^ designed a 2D-SIM system based on a diffraction grating and proposed the widely used generalized Wiener filtering SIM reconstruction method. In 2008, Gustafsson et al.^12^ designed a 3D-SIM system with true optical sectioning. The abovementioned methods follow the same processing procedure, in which high-resolution information is encoded into the illumination pattern and decoded through an inverse matrix method by acquiring three or five phase-shifted images in each focal plane. Here, we will take 2D-SIM as an example to explain the imaging process and reconstruction procedure.

In a 2D-SIM system, a total of nine images, including three orientations and three phases along each orientation, were acquired and assembled into a single reconstructed SR-SIM image to obtain isotropic resolution along the lateral directions^27^. In the Fourier domain, the multiplication sign ‘·’ and convolution sign ‘⊗’ in Eq. (2) become “⊗” and “·”, respectively, which can be expressed as:

5 $$\begin{array}{c}{D}_{\theta ,\varphi }(k)=[{\rm{S}}(k)\otimes {I}_{\theta ,\varphi }(k)]\cdot H(k)\\ =\frac{{I}_{0}}{2}[S(k)\cdot H(k)+\frac{1}{2}{e}^{-i\varphi }S(k-{k}_{\theta })\cdot H(k)+\frac{1}{2}{e}^{i\varphi }S(k+{k}_{\theta })\cdot H(k)]\end{array}$$

The symbol *H* (*k*) represents the Fourier transform of *h*(*r*), which is known as the OTF. Equation (5) indicates that the Fourier-shifted information $S(k-{k}_{\theta })$ and $S(k+{k}_{\theta })$ bring previously inaccessible information into the OTF for imaging, leading to an enhancement in the resolution of the optical system. By acquiring three spatial phase shifts in one illumination direction, a set of ternary linear equations can be constructed:

6 $$\begin{array}{c}{D}_{\theta ,{\varphi }_{1}}(k)=\frac{{I}_{0}}{2}[S(k)\cdot H(k)+\frac{1}{2}{e}^{-i{\varphi }_{1}}S(k-{k}_{\theta })\cdot H(k)+\frac{1}{2}{e}^{i{\varphi }_{1}}S(k+{k}_{\theta })\cdot H(k)]\\ {D}_{\theta ,{\varphi }_{2}}(k)=\frac{{I}_{0}}{2}[S(k)\cdot H(k)+\frac{1}{2}{e}^{-i{\varphi }_{2}}S(k-{k}_{\theta })\cdot H(k)+\frac{1}{2}{e}^{i{\varphi }_{2}}S(k+{k}_{\theta })\cdot H(k)]\\ {D}_{\theta ,{\varphi }_{3}}(k)=\frac{{I}_{0}}{2}[S(k)\cdot H(k)+\frac{1}{2}{e}^{-i{\varphi }_{3}}S(k-{k}_{\theta })\cdot H(k)+\frac{1}{2}{e}^{i{\varphi }_{3}}S(k+{k}_{\theta })\cdot H(k)]\end{array}$$

Integrating Eq. (6) into a matrix form, we obtain the following:

7 $$\begin{array}{c}\left[\begin{array}{c}{D}_{\theta ,{\varphi }_{1}}(k)\\ {D}_{\theta ,{\varphi }_{2}}(k)\\ {D}_{\theta ,{\varphi }_{3}}(k)\end{array}\right]=\frac{{I}_{0}}{2}M \left[\begin{array}{c}S(k)\cdot H(k)\\ S(k-{k}_{\theta })\cdot H(k)\\ S(k+{k}_{\theta })\cdot H(k)\end{array}\right]\\ M=\left[\begin{array}{c}1\frac{1}{2}{e}^{-i{\varphi }_{1}}\frac{1}{2}{e}^{i{\varphi }_{1}}\\ 1\frac{1}{2}{e}^{-i{\varphi }_{2}}\frac{1}{2}{e}^{i{\varphi }_{2}}\\ 1\frac{1}{2}{e}^{-i{\varphi }_{3}}\frac{1}{2}{e}^{i{\varphi }_{3}}\end{array}\right]\end{array}$$

Then, the three frequency information components $S(k)\cdot H(k)$, $S(k-{k}_{\theta })\cdot H(k)$, and $S(k+{k}_{\theta })\cdot H(k)$ can be separated by inverting the matrix *M*:

8 $$\left[\begin{array}{c}S(k)\cdot H(k)\\ S(k-{k}_{\theta })\cdot H(k)\\ S(k+{k}_{\theta })\cdot H(k)\end{array}\right]=\frac{2}{{I}_{0}}{M}^{-1}\left[\begin{array}{c}{D}_{\theta ,{\varphi }_{1}}(k)\\ {D}_{\theta ,{\varphi }_{2}}(k)\\ {D}_{\theta ,{\varphi }_{3}}(k)\end{array}\right]$$

**Fig. 4:**
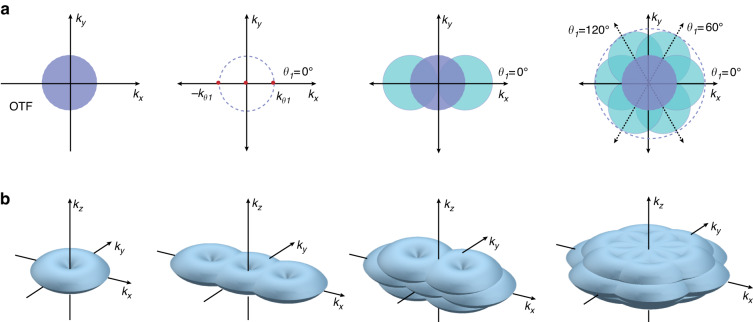
Schematic diagrams of the SIM reconstruction algorithms. **a** 2D-SIM reconstruction algorithm^11,27^ and **b** 3D-SIM reconstruction algorithm^12^. The frequency vector of the sinusoidal illumination pattern along the angular orientation *θ1* = *0°* is represented by ±*k*_*θ1*_. The observed frequency content of the structured illuminated specimen is a linear combination of the frequency content within the circular (2D-SIM) or torus-like shape (3D-SIM) regions. **a** © The IEEE

According to the Wiener deconvolution based on the minimum mean square error criterion^28,29^, these separated components are multiplied by the 2D-OTF, shifted back to their actual positions in Fourier space according to the illumination frequency, and then combined where they overlap. By changing the illumination angular orientation *θ* (typically, *θ*_1_ = 0*°*, *θ*_2_ = 60*°*, *θ*_3_ = 120*°*), and repeating the above procedure, all frequency content of the specimen within a circular region with a radius approximately twice that governed by the OTF of the optical system can be computed (Fig. 4a). Finally, the frequency sum is divided by the sum of the squares of the OTFs under different positions plus a small constant. The small constant is related to the SNR of the reconstructed image and approximates the inverse of the SNR. To smooth the reconstructed spectrum and suppress the ringing artifacts, the reassembled Fourier image is apodized with a cosine bell and then retransformed back to real space. Let ${\mathop{D}\limits^{\frown {}}}_{\theta ,m}(k+m{k}_{\theta })$ represent the separated frequency information components $S(k+m{k}_{\theta })\cdot H(k)$, where *m* = −1,0,1 is the three-component order at each illumination angle. The generalized Wiener filter function can be expressed as:

9 $$\mathop{S}\limits^{\frown {}}(k)=\frac{\sum _{\theta ,m}{H}_{m}^{\ast }(k+m{k}_{\theta }){\mathop{D}\limits^{\frown {}}}_{\theta ,m}(k+m{k}_{\theta })}{\sum _{\theta \text{'},m\text{'}}{|{H}_{m\text{'}}(k+m\text{'}{k}_{\theta \text{'}})|}^{2}+{w}^{2}}A(k)$$

Where $\mathop{S}\limits^{\frown {}}(k)$ is the estimate of the true sample frequency information *S*(*k*), and the sums are taken over three illuminating angular orientations *θ* and three component orders *m* at each orientation. *w*^2^ is the Wiener parameter, and *A*(*k*) is the apodization function. This reconstruction procedure can also be applied to 3D-SIM using a 3D-OTF instead of a 2D-OTF, as illustrated in Fig. 4b.

### SIM implementation modalities

#### OS-SIM implementation modalities

**Fig. 5:**
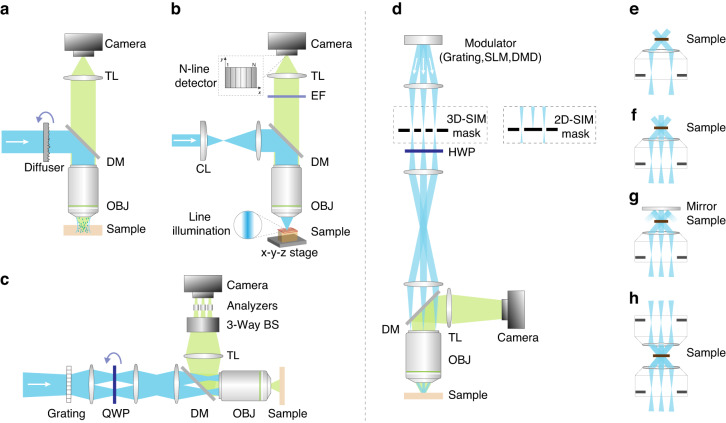
Diagram of SIM implementation modalities. **a** Hybrid speckle and uniform illumination microscopy (HiLo)^36^, **b** line-illumination modulation microscopy (LiMo)^43^, **c** polarization-illumination-coded structured illumination microscopy (picoSIM)^38,39^, **d** SR-SIM system diagram: **e** 2D-SIM^11^, **f** 3D-SIM^12^, **g** four-beam interference^47,48^, **h** six-beam interference^49^. **a** © The Optical Society, **b** © Nat Methods, **c** © The Phil Trans R Soc A

In addition to using a single spatial-frequency grid pattern, various other illumination patterns have been proposed: regular array of points^30^, square and hexagonal^31,32^, and dynamic speckle illumination (DSI)^33–35^. However, DSI microscopy is relatively slow because it needs several tens of images to generate an optically sectioned image of reasonable quality. To speed up DSI microscopy, Lim et al. developed a two-frame OS-SIM system that combines speckle and uniform illumination microscopy (Fig. 5a), also known as HiLo microscopy^36^. It is worth noting that this technology can be generalized to any type of patterned illumination, whether random (such as speckle) or nonrandom (such as a periodic grid or checkerboard pattern). In 2016, Philipp et al.^37^ proposed an adaptive HiLo microscope that uses an electrically tuneable lens, which can provide an axial scanning range of 1 mm with an axial resolution of approximately 4 μm and submicron lateral resolution. Additionally, several single-frame OS-SIM systems have been developed and improved.

For example, the idea of polarization-illumination-coded structured illumination microscopy (picoSIM) was proposed based on the homodyne OS-SIM concept, pioneered by Wicker and Heintzmann, and later realized by Appelt et al.^38,39^. In a picoSIM system (Fig. 5c), three individual light patterns are encoded in a single polarized illumination light distribution, allowing the acquisition of all SIM data needed for the computational reconstruction of a sectioned image in a single exposure. Recently, several improved technologies have been proposed for optical section imaging of uncleared thick tissues, including line-scanning SIM^40^, HiLo endomicroscopy^41^, and single-scan HiLo^42^. Among them, a single-scan HiLo method can obtain a wide-field image and its HiLo image in a single scan and is faster than the previous two methods for acquiring multiple thick tissue images. In 2021, based on a similar optical setup, Zhong et al.^43^ introduced another optical tomography method called line-illumination modulation microscopy (LiMo). This technology was further developed into fluorescent micro-optical sectioning tomography (fMOST) for whole-brain optical imaging. As depicted in Fig. 5b, the LiMo method can simultaneously record signals modulated by different intensities through multiline detection, enabling better background suppression. However, to reconstruct a one-line sample optical-sectioning image, LiMo needs at least two lines, whereas the minimum readout line number of the subarray mode is eight, limiting its maximum value to one-eighth of the detector throughput limit. In 2022, Fu et al.^44^ reported a 3D-resolved single-shot SIM system based on a digital micromirror device (DMD), a galvanometric mirror, and the HiLo algorithm. By synchronizing the DMD and galvanometer with an sCMOS camera, single-shot SIM can achieve optically sectioned imaging at a rate of 200 Hz and with lateral and axial resolutions of 0.41 and 1.93 µm, respectively.

#### SR-SIM implementation modalities

The axial resolution of wide-field fluorescence microscopy can also be improved by utilizing two opposite objective lenses, including standing wave illumination microscopy^16^ and I^5^M^21,45^. In these systems, the high-resolution axial information is encoded into the axially modulated structured illuminations generated by the interference of two opposing objective lenses. In addition, an I^5^M system utilizes two opposing objective lenses to illuminate and observe the sample from both directions, which results in no gaps in the effective OTF. Finally, a sevenfold improvement in axial resolution was achieved in 3D wide-field fluorescence microscopy by combining an I^5^M system with a truncated inverse filter followed by a few Jansson–van Cittert method iterations^46^.

Clearly, the lateral resolution can be improved using structured illumination patterns with lateral modulation generated by a diffraction grating^11,13^. Nonetheless, utilizing structured illumination patterns with either lateral or axial modulation can only improve the corresponding lateral or axial resolution, respectively, with no or limited effect on the other dimension. To avoid compromising between filling in missing cone information and maintaining resolution, 3D-SIM^12^ and its modifications have been proposed^47–51^. In 2008, Gustafsson et al.^12^ first designed a 3D-SIM system with true optical sectioning. As shown in Fig. 5d, three mutually coherent light beams interfere in the sample, forming a laterally and axially varying illumination pattern. 3D raw data are acquired with five pattern phases spaced by 2π/5, three pattern angles spaced 60° apart, and a focus step of 122 or 125 nm. The focal series of images are then processed utilizing the same generalized Wiener filtering procedure as 2D-SIM reconstruction but using 3D-OTF rather than 2D-OTF. Experimental results demonstrated that 3D-SIM can exceed the conventional resolution by a factor of two in each direction, resulting in a resolution of ~100 nm laterally and ~300 nm axially.

The limited and asymmetric range of light-gathering angles in a typical microscope is one factor that contributes to the axial resolution being several times worse than the lateral resolution. To address this issue, Shao et al.^49^ developed an I^5^S system by combining a 3D-SIM structured illumination system with the two opposing objective lens geometries of an I^5^M system (Fig. 5h), achieving an ~100 nm spatial resolution in all three dimensions. In 2020, an experimental I^5^S system setup was simplified using a low NA and high working distance objective^47^. In 2022, Li et al.^48^ placed a mirror directly opposite the sample and realized four-beam interference (Fig. 5g) with higher spatial frequency components than 3D-SIM, resulting in a lateral resolution of ~120 nm and an axial resolution of ~160 nm, producing nearly isotropic reconstructions. Additionally, in 2022, Alexandr et al.^51^ proposed a moving fringe SIM (MF SIM) method, which uses a two-beam illumination pattern varying along the optical axis instead of the standard three-beam illumination pattern to avoid restricting the fringe pattern spatial frequencies.

Another limitation of traditional SIM systems is the time-consuming mechanical manipulation of the diffraction grating, which makes it challenging to observe living cells in real-time. To address this, researchers have explored various methods to enhance SIM imaging speed with the rapid development of optical system hardware. For example, many optical systems now utilize spatial light modulators (SLM) or DMD rather than physical grating^52–57^ to improve speed and flexibility. Other approaches include the instant SIM (iSIM) system, which uses optical rather than digital image-processing operations^58,59^; multifocus SIM, which employs a multifocus diffractive optical element^60^ to capture multiple focal planes simultaneously; and galvanometer set^61,62^ or electro-optics modulator (EOM)^63^ - based SIM systems.

### Nonlinear SIM

**Fig. 6:**
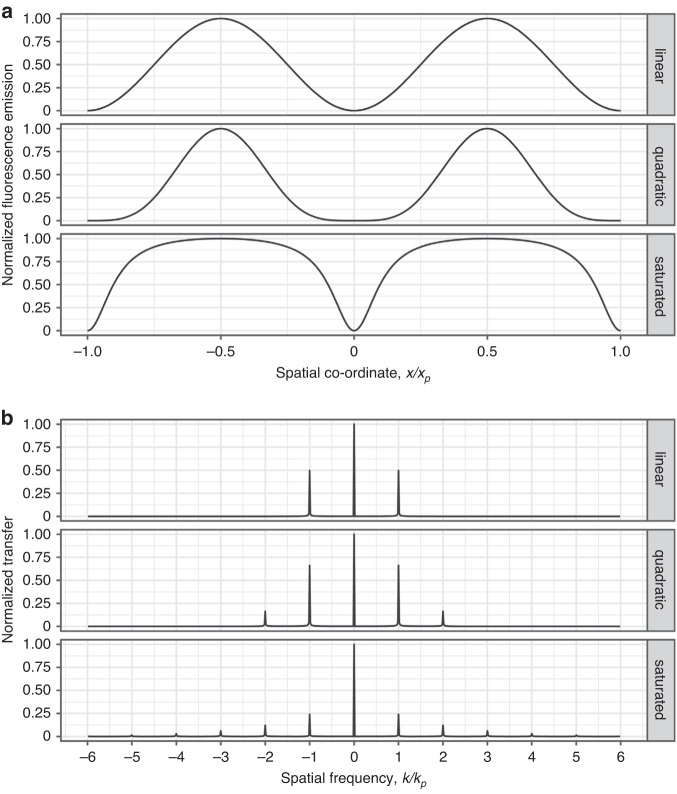
Comparison of nonlinear and linear SIM. **a** Comparison of linear and nonlinear SIM methods in terms of fluorescence emission in the spatial domain. The top row shows linear SIM, the middle row shows nonlinear SIM with quadratic nonlinearity, and the bottom row shows nonpolynomial SIM with fluorescence saturation. **b** The Fourier transform of **a**. The pattern period and spatial frequency are denoted by *x*_*p*_ and *k*_*p*_, respectively^137^. **a**, **b** © The Phil Trans R Soc A

As the illumination patterns of a linear SIM system are also fundamentally limited by the Abbé diffraction limitation, the final resolution extension is at most twofold that of wide-field microscopy. However, this barrier can be overcome by using more sophisticated optical schemes or by fundamentally exploiting nonlinear sample responses. In NL-SIM, the nonlinear illumination intensity frequency is not limited by the optical microscope’s NA. Similar to the traditional linear SR-SIM reconstruction process, the generalized Wiener filtering reconstruction method^64,65^ is also used for NL-SIM. However, as shown in Fig. 6, NL-SIM generates more high-order harmonics, requiring more raw images to decompose the high-resolution sample information. For the highest harmonics that are not negligible, the modulation amplitude is too low to produce reliable results. Therefore, the theoretical complex amplitude values are used as weighting values for the junction of the Fourier information components^56,66^. A series of NL-SIM implementations are summarized and classified as follows:

#### Category 1: saturated SIM

**Fig. 7:**
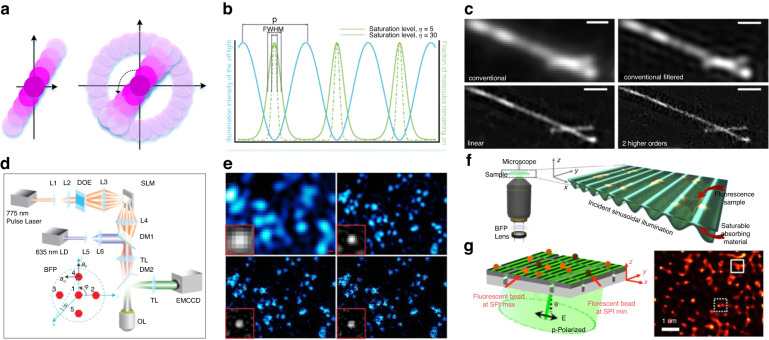
NL-SIM Implementation modalities. **a** Saturated SIM^64^ Left: Observable regions for conventional microscopy (dark purple), linear structured illumination (purple), and nonlinear structured illumination microscopy (light purple) based on the three lowest harmonics. Right: The corresponding observable regions when the procedure was repeated along the other 11 pattern orientations. **b**, **c** Nonlinear SIM based on a fluorescent photoswitchable protein^68^. **b** Real-space representation. Spatially patterned off light (blue) drives the molecules to the off state. In a small region surrounding the zeros of this pattern, a fraction of the molecules will remain in the state (green). At a high saturation level (dashed green), only those molecules falling directly in the pattern’s zero will remain fluorescent. **c** Two Dronpa-coated microtubules were imaged using conventional TIRF, conventional filtered TIRF, linear SIM-TIRF, and NL-SIM-TIRF with two higher-order harmonics. Scale bar, 500 nm. **d** Diagram of 3D SI-STED microscopy^75^. The 635 nm excitation beam generates wide-field illumination on the image plane. The 775 nm depletion beam generates 3D SI on the image plane by phase modulation on the Fourier plane using a spatial light modulator (SLM). **e** Live-cell nonlinear SIM based on patterned photoactivation^56^. Caveolae in a COS7 cell at 23 °C transfected with Skylan-NScaveolin, comparing TIRF with deconvolution (top left, 220-nm resolution), TIRF SIM (top right, 97-nm resolution), PA NL-SIM (bottom left, 62-nm resolution), and saturated PA NL-SIM (bottom right, 45-nm resolution). **f** SAN-SIM^72^ Left: Graphical representation of the illumination configuration; Right: Magnified view of the conversion scheme of the illumination pattern. **g** Demonstration of PSIM resolution improvement with closely located beads^71^. **a**–**c** © The Proc Natl Acad Sci USA, **d**, **f** © The Optical Society, **e** © Science, **g** © The NANO Letters

Heintzmann et al.^65^ first developed the saturated SIM theory in 2022, which uses fluorescence saturation as a nonlinear process to relate emission to excitation. They simulated a 2D extension of the nonlinear patterned excitation technique and discussed methods to substantially reduce the number of required raw images^67^. In 2005, Gustafsson^64^ experimentally demonstrated the saturated SIM concept and achieved <50 nm 2D point resolution on dye-filled polystyrene beads using a total of 108 raw images (Fig. 7a). While saturated SIM can theoretically yield an infinite resolution, in practice, its resolution is limited by factors such as the SNR and photostability “soft” materials. In addition, extremely high light intensity is required to saturate SIM, which can accelerate photobleaching and even damage fixed tissues, thus limiting its application in the study of biological samples.

#### Category 2: nonlinear SIM based on fluorescent photoswitchable proteins

In 2012, Rego et al.^68^ discovered that the reversible photoswitching (Fig. 7b, c) of the fluorescent protein Dronpa can provide the desired nonlinearity at light intensities six orders of magnitude lower than those required for saturated SIM. Using ultralow light powers, they demonstrated a resolution of ~40 nm on purified microtubules. In 2015, Li et al.^56^ proposed a technique called patterned activation of photoswitchable fluorophores (PA NL-SIM) that improved the resolution of live-cell SIM to a range of 45–62 nm using approximately 20–40 frames of raw images (Fig. 7e). Additionally, they combined PA NL-SIM with lattice light-sheet microscopy to observe the entire volume of whole cells in 3D, achieving an axial resolution fivefold better than that of conventional wide-field microscopy. However, the digital state of individual molecules in PA NL-SIM is either off or on, which leads to hyper-Poisson noise and can degrade the quality of the SIM reconstruction. Therefore, there is a need to synthesize novel fluorescent dyes that can tolerate large numbers of on-off cycles and improve the performance of PA NL-SIM, as anticipated by researchers^69^.

#### Category 3: nonlinear SIM based on surface plasmons

In 2010, Wei et al. proposed a method called plasmonic SIM (PSIM) that combines SIM with tuneable surface plasmon interferometry to achieve the desired nonlinear illumination pattern^70^. Simulation results showed that the PSIM technique can achieve imaging resolutions that are three- and four-fold higher than those of conventional wide-field images. In 2014, they experimentally demonstrated the unique properties of PSIM^71^, as illustrated in Fig. 7g. However, it is important to note that PSIM has limitations. It can only form high-resolution images of samples that are near the metal surface. Additionally, the fluorescence efficiency of PSIM is lower than that of conventional SIM, which may limit its practical utility in some applications. In 2022, Samanta et al. proposed a new technique called saturable absorption-assisted nonlinear SIM (SAN-SIM) by exploring the saturable absorption property of an absorbing material^72^ (Fig. 7f). They demonstrated that SAN-SIM can achieve a resolution that is more than twofold higher than the diffraction limit without the need for high-power illumination or specific fluorescent dyes.

#### Category 4: nonlinear SIM based on stimulated emission depletion (STED)

This method is considered suitable for live-cell imaging due to its speed and minimal invasiveness^64^. One example is the surface plasmon resonance (SPR)-enhanced STED-SIM^73^ method, which enables high-speed imaging at 30 nm resolution over a >100 µm^2^ area with single-molecule sensitivity. However, this method requires complex sample preparation, and only the sample surface can be observed. Thus, it may not be suitable for certain types of samples or applications that require imaging of deeper regions within the sample. In 2015, Dake et al.^74^ proposed a method called structured-excitation STED-SIM (SSTED-SIM), which can improve the optical resolution of the widefield of view to approximately λ/7 and reduce the background fluorescence signal. In 2018, Xue et al.^75^ developed a 3D SI-STED microscope that uses five coherent beams to interfere and generate a 3D grid depletion pattern (Fig. 7d). The 3D SI-STED technique enables “simultaneous” 3D superresolution imaging over a volume with 60 nm lateral resolution and 160 nm axial resolution at a 5 Hz imaging rate. Additionally, this technique can significantly reduce photobleaching and photodamage, making it suitable for long-term imaging of live cells and tissues. Notably, in the SI-STED method, the high-order spatial frequency components contained in the effective emission region are related to both the illumination pattern period and the depletion power. This means that the effective emission region is decomposed by a series of impulse functions rather than sinusoidal waves. As a result, a correspondingly weighted summation of the intermediate images is performed in the spatial domain, rather than generalized Wiener filtering. This unique approach allows for more accurate and precise image reconstruction.

## Development of SIM reconstruction methods

### Development of OS-SIM reconstruction methods

**Fig. 8:**
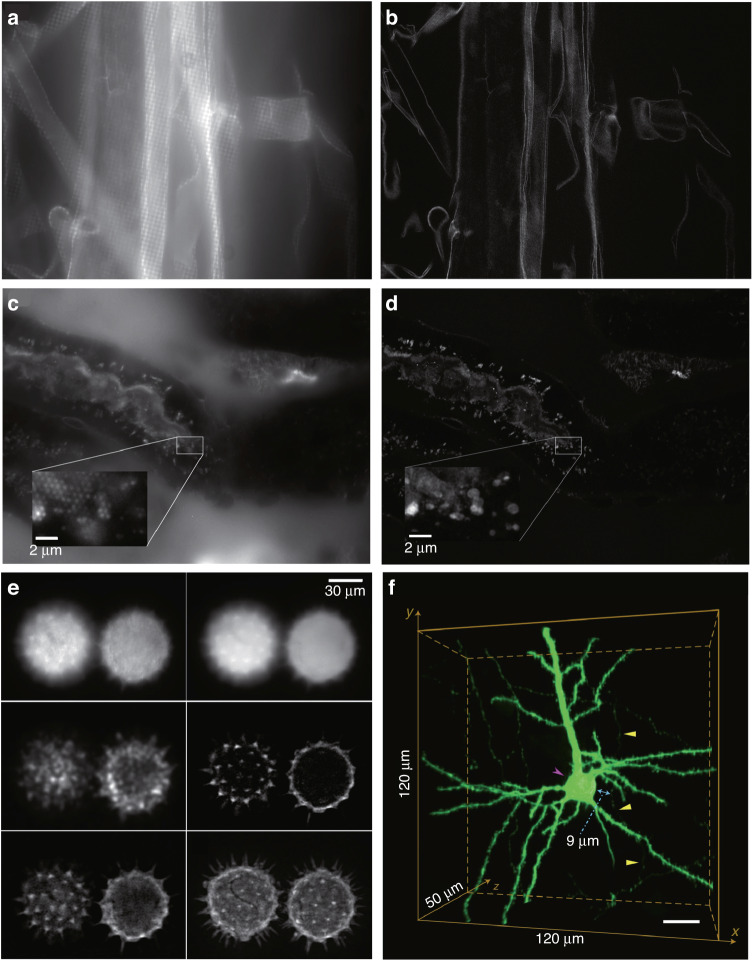
Optical section images reconstructed under different OS-SIM algorithms. **a**–**d** Comparison between single phase images illuminated by square **a** or hexagonal **c** illumination patterns and quasiconfocal images **b** or **d**^31^. **e** HiLo technique applied to a pair of fluorescent pollen grains located at slightly different depths^36^. Top row: speckle-illumination image (left), uniform illumination image (right); middle row: intermediate low-pass image (left), intermediate high-pass image (right); bottom row: composite full resolution optically sectioned image (left), extended focus image obtained from a maximum intensity projection of 80 slices separated by 0.5 μm steps. **f** Whole-brain imaging of the anterograde projections of AAV-YFP-labelled neurons in the motor cortex^43^. **a**–**d** © The Journal of Microscopy, **e** © The Optical Society, **f** © Nat Methods

In addition to commonly used RMS algorithms, there are other OS-SIM reconstruction methods, including projection (i.e., sum, maximum, and super-confocal), scaled subtraction of the out-of-focus estimation, and a modified version of Fourier-space treatment (known as patterned excitation microscopy processing). Heintzmann et al.^30^ discussed and analysed these three strategies based on a regular array of point illumination patterns. Simulation and experimental results demonstrated that while projection methods, especially the super-confocal method, have exceptional optical sectioning characteristics, they also exhibit more residual patterning than the other two methods. PEM processing can provide high resolution but is computationally expensive. In 2014, Schropp & Uhl^31^ pointed out an alternative structured illumination microscopy employing square or hexagonal illumination patterns (Fig. 8a–d). Rather than shifting a regular array of point illumination patterns row by row along the x- and y-axes, they shifted 2D illumination patterns unidirectionally along pattern-dependent angles. This approach results in an isotropic power spectral density and opens new possibilities for high-resolution imaging in biological and materials science applications.

To streamline the measurement setup and enhance imaging speed, a two-frame OS-SIM algorithm was proposed, such as the HiLo algorithm^36^ or the amplitude demodulation algorithm based on the Hilbert-Huang transform^76,77^. HiLo microscopy employs a nonuniform (fixed-frequency or speckle) image to provide low-resolution information with spatial frequencies below a user-specified cut-off frequency, while a uniform illumination image is acquired to provide high-resolution information with a spatial frequency above the cut-off frequency. By appropriately setting the cut-off frequency and fusing the low- and high-resolution information, a full-resolution optically sectioned image can be recovered (as shown in Fig. 8e). However, in the HiLo algorithm, additional attention must be paid to the process of fusing the low- and high-resolution information. Moreover, the amplitude demodulation method based on the Hilbert-Huang transform necessitates two mutually π phase-shifted raw structured images. However, the imaging speed of this two-frame OS-SIM method is still constrained by the camera’s capabilities (~34 fps). Subsequently, a single-frame OS-SIM algorithm was proposed. In 2019, Wang et al.^78^ proposed a Fourier bandpass filtering algorithm to reconstruct optical section images by shifting the in-focus signals to the +1st order in the Fourier domain. However, this method requires perfect separation of Fourier spectrum components using a bandpass filter, which can reduce the lateral resolution. In 2021, Zhong et al.^43^ further developed a high-definition fluorescent micro-optical sectioning tomography (HD-fMOST) method for whole-brain optical imaging with submicrometer-voxel resolution, based on the LiMo method (Fig. 8f).

In addition, deep learning has demonstrated its effectiveness in OS-SIM techniques. It can be used to solve issues such as high computational costs and oversimplification of optical systems for some deconvolution techniques^79,80^ (such as Wiener filtering^81^ and Richardson–Lucy (RL) deconvolution^82,83^). For example, in 2018, Zhang et al.^84^ developed a deep learning-based computational algorithm, which only requires a single wide-field image and a corresponding optical sectioning reference image to train a convolutional neural network (CNN). This algorithm can reconstruct optical section images with lower noise, fewer artifacts, and higher imaging depth at an optimized frame rate of 14 Hz. In 2021, Chai et al. proposed a one-shot optically sectioned method called Deep-OS-SIM^85^, which is based on deep-learning techniques. Unlike other methods that use low entropy wide-field images, this approach takes full advantage of the high entropy properties of structured illumination images to train a CNN model. Optical-sectioning imaging using this method only requires a single image for decoding, thereby improving the raw image acquisition efficiency by 50% compared to the two-frame HiLo method. However, similar to other deep learning-based methods, this method requires expertise in deep learning, and it is currently restricted to specific projected illumination patterns and samples for OS-SIM. If the statistical characteristics of samples or the illumination pattern modes change, retraining for variations in imaging parameters will be necessary.

### Development of 2D-SIM reconstruction methods

#### Parameter estimation

SR-SIM reconstruction is essentially an ill-posed inverse problem. As mentioned in the subsection “Basic SR-SIM algorithm”, solving and separating the spectra of the sample and then moving them back to their correct positions is crucial during SIM reconstruction. This process requires precise knowledge of structured illumination patterns, especially in those techniques that rely on high-order harmonics to improve resolution^86,87^. Even slight deviations in the reconstruction parameters from the correct ones can lead to noticeable artifacts in the reconstructed images, such as ghosting and fringing.

The periodic illumination pattern parameters include the illumination frequency vector, angle, phase, and modulation depth. Although the illumination frequency vector can be determined with high precision and reproducibility using structured illumination generators such as SLM^52–54^ and DMD^55,88^, the initial phase is difficult to determine accurately without prior knowledge. Moreover, factors such as sample movement, system-dependent optical aberrations, and photobleaching can cause the pattern position to shift in the raw images, making it challenging to estimate the parameters based on prior knowledge. As a result, numerous algorithms have been proposed for postprocessing parameter estimation of periodic illumination patterns.

**Fig. 9:**
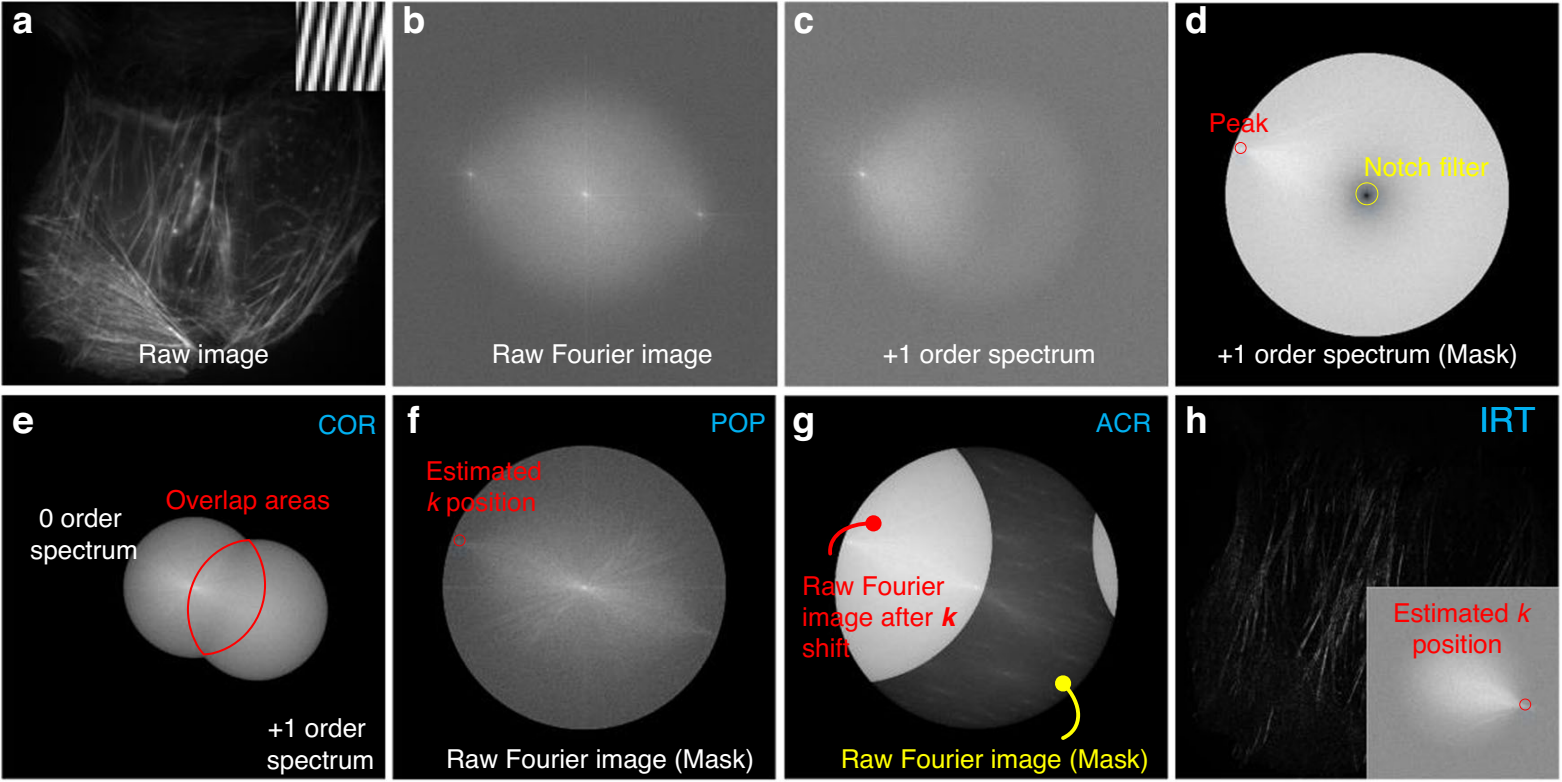
Parameter estimation in 2D-SIM. **a** Raw image of actin from a homemade sample U2OS cell acquired using a 2D-SIM system. **b** The corresponding Fourier spectrum distribution of (**a**). **c** One of the separated spectra, +1 order spectrum. When estimating the frequency vector *k*, there are two steps: **d** Rough estimation^91^. Combined with a notch filter, the peak position in the +1 order-separated spectrum is extracted; **e** Precise estimation^12^. The cross-correlation between the separated 0- and +1 order spectra is maximized. There are four methods when estimating the initial phase φ: **e** COR^94^. Sum the pixel values of the overlap areas between the separated 0 and +1 order spectrum, and extract the angle of the complex factor obtained by summation; **f** POP^92,93^. Directly extract the phase at the peak in the raw Fourier image; **g** ACR^95^. Sum the overlapping areas of the autocorrelation of the respective raw Fourier images; **h** IRT^96^. Sum the raw images with phase shift, transform the summation into Fourier space, and then extract the phase of the estimated peak

Based on the work of Gustafsson et al.^12^, the frequency vector can be retrieved by iteratively maximizing the cross-correlation (COR) of the overlap areas between the first separated and zero orders, assuming an equidistant phase distribution (Fig. 9e). The modulation depth and phase offset can then be obtained by calculating the absolute value and angle of the complex factor of the overlap areas, respectively. One advantage of this method is that the separation of orders only relies on the relative phase between individual images. To speed up the parameter estimation process, a notch filter (Fig. 9d) was later introduced to roughly extract the peak position of the +1 order-separated spectrum before optimizing the maximum cross-correlation^89–91^.

In many cases, residual orders may exist in the spectra separated by unmixing due to imprecise individual illumination phases, which can be minimized by estimating the phase offset of each illumination pattern. Shroff et al.^92,93^ proposed a Fourier domain phase of the peak (POP) estimation method without prior knowledge of the phase shifts, which is suitable for live-cell imaging, as shown in Fig. 9f. However, this method is inappropriate for high-frequency illumination patterns such as those in TIRF mode. As the phase information is directly estimated from the raw image, three conditions should be simultaneously met: the raw image has a high SNR, the high-frequency component decays rapidly enough, and the illumination pattern frequency is lower than the cut-off frequency determined by the support area for OTF detection. In 2013, Wicker et al.^94^ developed an iterative optimization method to determine the pattern phases using the COR between separated components in cases where the illumination pattern was too fine to detect. Although this method can robustly determine the relative pattern phase in SIM raw images with a precision below λ/100, its iterative nature inevitably results in longer computation times. In a later study, Wicker^95^ presented a faster and more robust noniterative autocorrelation reconstruction (ACR) method for determining a pattern’s phase, as shown in Fig. 9g. This method calculates each illumination pattern’s phase from the autocorrelation of its corresponding raw Fourier image and typically achieves precision less than λ/500 at realistic SNR levels. In 2016, Lal et al.^27^ provided a comprehensive theoretical overview of 2D-SIM algorithms, including determining the illumination frequency vector using the ACR method and estimating the phase offset through iterative optimization of the correlation function between the illumination pattern and the sample’s Fourier image.

**Fig. 10:**
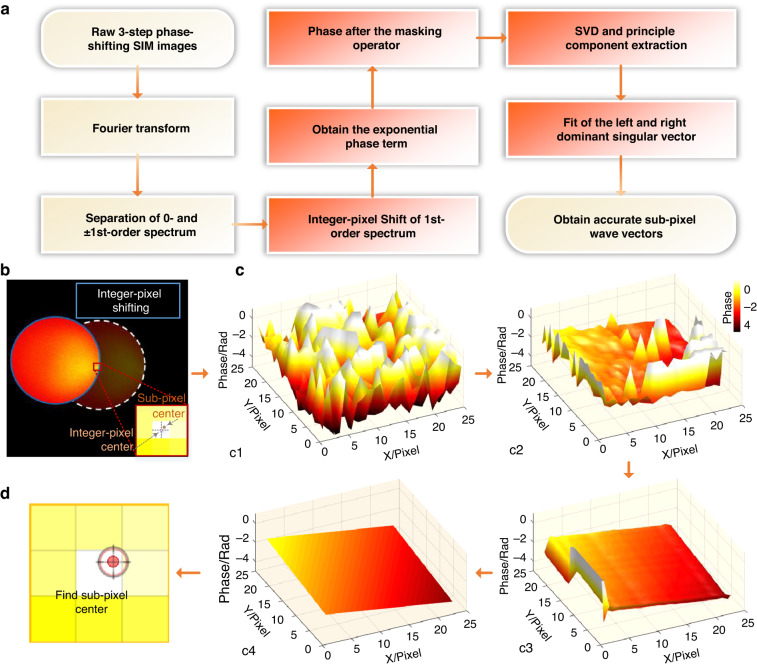
Schematic diagram illustrating the PCA-SIM estimation algorithm. **a** Flow chart of the parameter estimation in PCA-SIM^100^. **b**–**d** Output results of red boxes in **a**. **b** The first-order spectrum after shifting the integer-pixel frequency vector. **c** The phases of the phasor matrix obtained from **b** after different operations: the original phases c1, phases after applying the masking operator c2, phases after applying PCA c3, and phases after least-squares fitting c4. **d** Obtained frequency vector with subpixel accuracy

In 2016, Zhou et al.^96^ proposed an image recombination transform (IRT) algorithm (Fig. 9h), which utilizes the phase difference among three raw images to obtain a high-precision initial phase. By combining this algorithm with their own DMD-projection-based, multicolor LED-illumination SIM system, they achieved low excitation intensity fluorescence imaging even less than 1 W∕cm^2^. However, the IRT algorithm only considered a phase shift of π/2 and two orientations separated by 90°, limiting its application in general scenarios. To overcome this problem, Zhao et al.^97^ reported an enhanced IRT algorithm that can handle arbitrary phase shifts. It should be noted that the POP, ACR, or IRT algorithms cannot guarantee accurate phase estimation when the raw images have low SNR or weak modulation depth. In addition, for certain periodic samples, the ACR algorithm requires the modulation vector to be distinct from the spatial frequency vector. To address these issues, Cao et al.^98^ proposed a noniterative phase estimation method based on an inverse matrix by incorporating extra matrices into the phase estimation algorithm. However, since the parameters of the inverse matrix can affect the phase estimation error, it is essential to select an appropriate parameter set to decrease the average phase error, which lacks objectivity. In 2022, Qian et al.^99,100^ introduced principal component analysis (PCA) into SIM for the first time to identify the frequency vectors and pattern phases of the illumination pattern (Fig. 10). They demonstrated that PCA-SIM can achieve fast and accurate noniterative parameter estimation (with frequency vector precision below 0.01 pixels and relative phase precision of 0.1% of 2π under typical noise levels) that is also robust at low SNRs. This allows for real-time superresolution imaging of live cells in complicated experimental scenarios.

#### Fourier domain reconstruction algorithms

FDR algorithms, also known as direct methods, are the most used methods for SIM reconstruction. As mentioned earlier, the first proposed FDR algorithm was the generalized Wiener filtering algorithm. However, this algorithm is not only affected by imprecise parameter estimation but also vulnerable to systematic aberrations and the SNR of raw images^101^. In addition, its reconstruction speed does not meet the requirements of real-time imaging. To address these issues, various improved FDR algorithms have been proposed, which can be classified into the following three parts.

##### Part 1: SIM reconstruction for suppression of optical aberrations

Optical aberrations, such as systematic spherical aberration and sample-induced aberrations, not only cause artifacts, loss of resolution, and reduced image contrast in SIM-reconstructed images but also limit the technique’s application to samples thinner than a single cell^102,103^. Even small optical aberrations, which have minimal influence on a diffraction-limited image, can cause severe artifacts in SIM-reconstructed images. Moreover, the degree of image distortion caused by spherical aberration is influenced by a range of physical parameters, including cover glass thickness, a refractive index of the sample embedding medium/immersion oil, and sample temperature, all of which are empirical and add to the complexity of the problem. Typically, there are two physical ways to improve spherical aberration: choosing an appropriate immersion oil or adding adaptive optics in the imaging path^87,104–107^. In recent years, algorithms have been proposed to improve SIM imaging quality, including PSF engineering^91,108^ and tiled reconstruction methods^109,110^.

In 2016, Perez et al.^108^ proposed an RL-based deconvolution^111^ filtering step for both raw and reconstructed images. This method depends only on unbiased filtering steps during reconstruction, without requiring any parameter tuning. However, it does not suppress the effects of out-of-focus background and spectral inhomogeneity on the reconstructed image. In 2020, Wen et al.^91^ presented a high-fidelity SIM (HiFi-SIM) reconstruction algorithm, which engineers the effective PSF into an ideal form. By combining a normalized cross-correlation method with a spectrum notch, HiFi-SIM can automatically estimate the illumination pattern parameters. Furthermore, it can effectively reduce common artifacts without sacrificing delicate structures and improve axial sectioning for samples with a strong background.

**Fig. 11:**
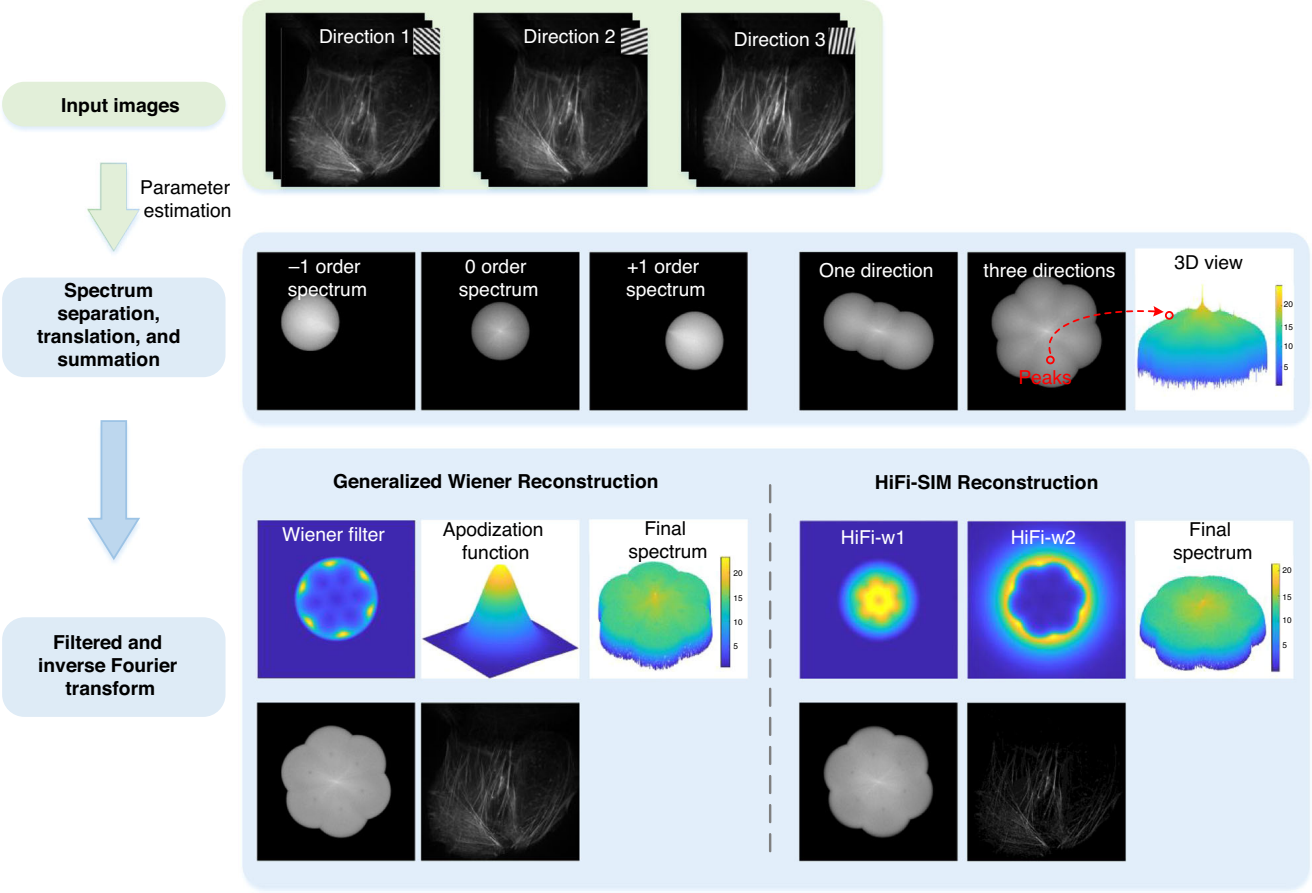
Fourier domain-based SIM reconstruction methods. The 2D superresolution SIM reconstruction process using two different Fourier domain reconstruction methods: the generalized Wiener filter (OpenSIM)^27^ and high-fidelity SIM (HiFi-SIM)^91^

Here, we present schematic diagrams of two FDR reconstruction procedures, the generalized Wiener filtering method (OpenSIM)^27^ and HiFi-SIM (Fig. 11). Apparently, the spectrum reconstructed by HiFi-SIM is flatter and smoother than that reconstructed by OpenSIM. Additionally, the reconstructed image produced by HiFi-SIM has higher contrast while preserving details.

In 2020, Hoffman et al.^109^ developed a tiled reconstruction method to achieve artifact-free whole-slide imaging with a large field-of-view in SIM, which can alleviate many common SIM reconstruction artifacts caused by global parameter estimation errors. In this method, each raw image was divided into overlapping tiled subsets, and each subset was reconstructed using independently measured or user-optimized parameters. These subsets were then reassembled into a composite superresolution image covering the original field of view. Furthermore, Johnson et al.^110^ proposed a Bayesian estimation-based SIM reconstruction method that combined SIM with image-stitching and devignetting methods to provide artifact-free stitched images with optical sectioning and superresolution properties. The results of five different samples demonstrated that the stitched SIM images were useful for intraoperative histology.

##### Part 2: SIM reconstruction under a low SNR situation

When acquiring raw SIM images, using a higher signal level can result in better-quality reconstructed images. However, this can accelerate sample photobleaching and limit the number of time points for live-cell images. On the other hand, acquiring raw images at low signal levels can result in considerable noise, leading to artifacts in the reconstructed image. To minimize these artifacts, the parameters in the Wiener filter^27^ are typically set manually, which is user-dependent and lacks objectivity.

Subsequently, a series of regularization-based iterative optimization methods were proposed based on the prior knowledge of structured illumination patterns. For example, in 2014, Chu et al.^112^ introduced a total variation (TV) constraint for SIM reconstruction. The algorithm can image at least 15 times more time points than a traditional Wiener filtering reconstruction method. However, the reconstructed image contains stepped artifacts due to the overcorrection of edge information. Lukeš et al.^113^ proposed a SIM method based on the maximum a posteriori (MAP) probability. Combined with homodyne detection, this method can suppress out-of-focus information, improve spatial resolution, and enable the reconstruction of 2D and 3D images of cells, even with weak signals. They later developed an open-source, modular function set, SIM-Toolbox for MATLAB, which supports OS-SIM and SR-SIM image reconstruction^114^.

In 2018, Huang et al.^115^ reported a deconvolution method for SIM, called Hessian-SIM, which utilized prior knowledge of the continuity of multidimensional biological structures based on Hessian matrices. This method enabled ultrafast live-cell superresolution imaging (such as structural dynamics of mitochondrial cristae) with a spatiotemporal resolution of 88 nm and 188 Hz. Moreover, compared with TV-SIM, Hessian-SIM can retain more image details while reducing noise. In the same year, Boulanger et al.^116^ proposed a nonsmooth convex optimization method for SIM reconstruction. However, this method requires heavy computation and takes a long time to converge. In 2020, Yu et al.^117^ implemented a second-order optimally regularized SIM (sorSIM) method, which utilizes second-order partial derivatives to suppress the stepped artifacts that appear in TV-SIM. This method achieves a balance between resolution enhancement and noise immunity. In 2021, Zhao et al.^118^ added the sparsity of biological structures to Hessian-SIM and proposed a Sparse-SIM deconvolution algorithm, which can achieve a resolution of ~60 nm at a frame rate of up to 564 fps. This method also enables four-color, 3D live-cell superresolution imaging at ~90 nm resolution. However, the resolution enhancement of Sparse-SIM will depend on factors such as the image SNR and optimal parameter selection, which can be cumbersome for different biological samples. In 2022, Zhou et al.^119^ established a nonuniform sCMOS noise model and proposed a corresponding noise-corrected SIM reconstruction algorithm based on the stable biconjugate gradient descent algorithm (Bi-CGSTAB)^120^ and split Bergman algorithm^121^. Simulation results indicated that this noise-corrected SIM reconstruction algorithm can effectively suppress sCMOS noise-related reconstruction artifacts. Recently, Hou et al.^122^ developed an MRA deconvolution algorithm for fluorescence images, which uses framelet and curvelet domain sparsity to regularize the solution. This algorithm allows fine detail recovery even with a negative SNR and provides more than twofold physical resolution enhancement with fewer artifacts than maximum likelihood estimation (MLE) methods. Furthermore, they developed a DeepMRA deconvolution algorithm, which can address severer backgrounds and better preserves high-frequency and low-intensity details that are commonly disrupted by other algorithms.

##### Part 3: SIM reconstruction speed improvement

To enable real-time observation for live-cell imaging, various attempts have been made to enhance the SIM imaging speed. In addition to improvements in optical system hardware, several algorithm optimizations have been proposed, such as frame reduction of raw images, rolling reconstruction, and GPU acceleration.

Preliminary results have demonstrated that a superresolution image can be reconstructed from four raw images^123,124^. In 2017, Ströhl and Kaminski proposed acquiring three raw images under different illumination orientations, and indicated that the frame rate can be doubled by using the joint RL deconvolution algorithm^125^. However, in 2018, Lal et al.^126^ found only approximately a 1.5× resolution enhancement in the final image reconstructed using three raw images and concluded that at least four raw images are required to double the resolution. In 2022, Zeng et al.^127^ introduced polarization modulation to the frame reduction imaging model, proposing a complete and versatile imaging model called PRSIM. They indicated that for polarized samples, polarization-related artifacts can be reduced by combining a Fourier domain iterative reconstruction algorithm. However, these frame reduction methods rely on assumptions about the image-formation process, and the final reconstructed results are limited by the type of noise.

In 2017, Ma et al.^128^ proposed the combination of SIM with an interleaved reconstruction strategy (SIMILR) to maximize the use of each subframe of the acquisition series. This method enabled the observation of highly dynamic structures, such as the endoplasmic reticulum, which undergoes continuous rapid growth or shape changes. Later, in 2018, Guo et al.^129^ employed SIMILR in grazing incidence SIM, which utilizes highly inclined laminar illumination^130^. This method achieved dynamic imaging of events near the basal cell cortex at 97 nm resolution and 266 fps over thousands of time points. In addition, combining Hessian-SIM with a ‘rolling’ reconstruction procedure allowed for a maximum number of frames in time-lapse imaging of up to 291 fps^115^. Similarly, Sparse-SIM achieved a frame rate of 564 fps^118^.

In terms of GPU acceleration, various SIM reconstruction methods have been developed using programming languages such as CUDA C++, Java, and Python^89,131–133^. For example, in 2019, Markwirth et al.^132^ proposed a video-rate immediate GPU-accelerated open-source reconstruction (VIGOR) method by recreating, modifying, and extending the fastSIM^134^ approach and image reconstruction software. The results demonstrated that multicolor SR-SIM can be reconstructed at video frame rates (25 reconstructed fps or more), with a delay of less than 250 ms between measurement and reconstructed image display. In 2021, Gong et al.^131^ presented a GPU-accelerated SIM method using a hexagonal illumination pattern based on the Python language. This method can process over 239 input raw images (512 × 512 pixels) per second and generate over 34 superresolution frames per second at 1024 × 1024 pixels. However, it should be noted that in these GPU acceleration methods, the illumination parameters are estimated and calibrated in advance (e.g., using the COR algorithm) and then reused in the subsequent reconstruction, making it challenging to address complex dynamic operating environments, such as artificial interference and environmental perturbations, which can lead to drift in illumination patterns.

#### Spatial domain reconstruction

**Fig. 12:**
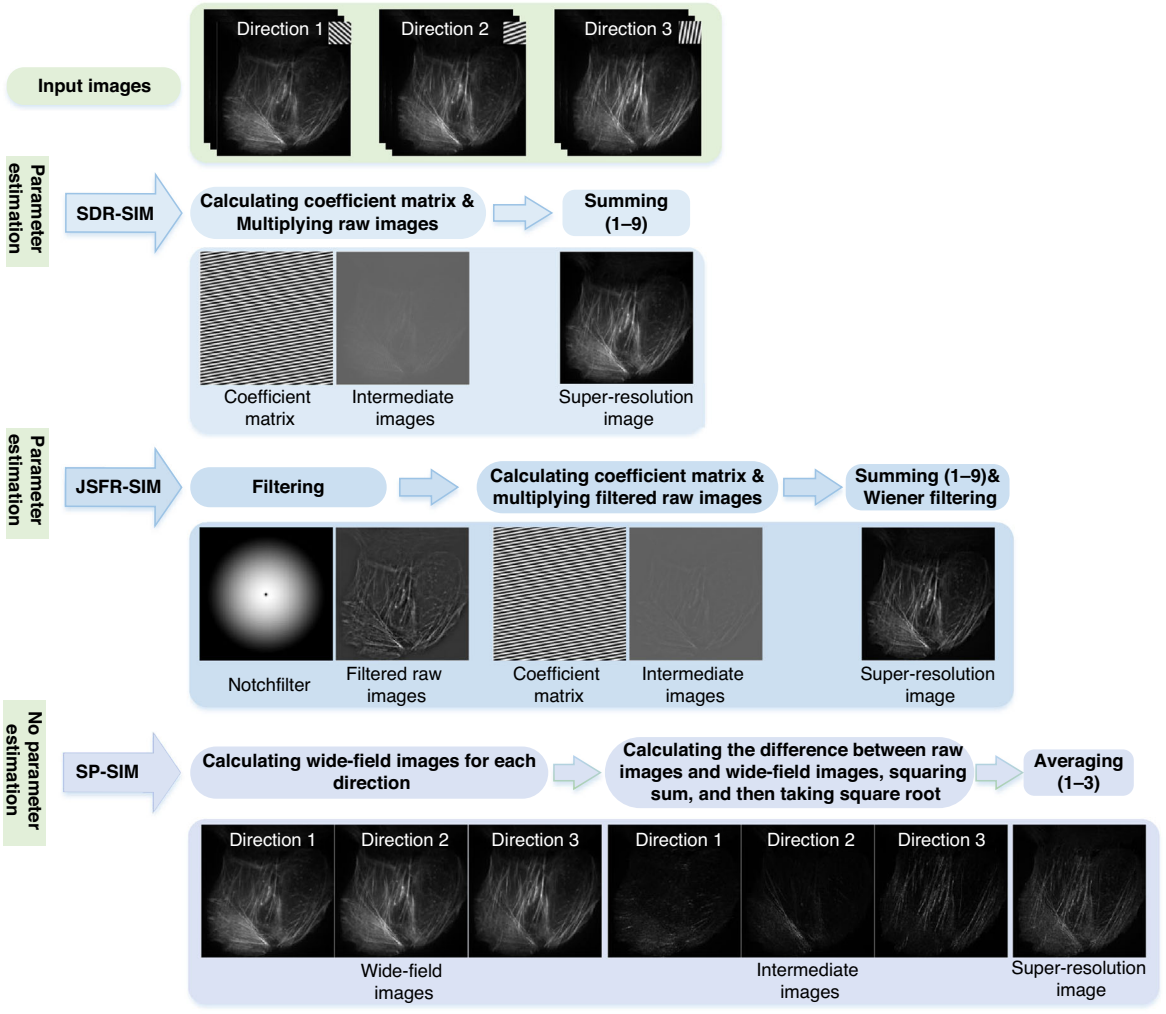
Spatial domain-based SIM reconstruction methods. The process of reconstructing SIM in the spatial domain involves several methods, including: spatial domain reconstruction SIM (SDR-SIM)^137,138^, joint space and frequency reconstruction SIM (JSFR-SIM)^139^, and shifting phase SIM (SP-SIM)^141^

The SDR approach was initially proposed by Cragg & So during the development of SIM^135,136^. They created an enhanced 2D image by taking images at different phases and directions of a structured illumination pattern and forming a weighted sum. SDR requires the same number of raw images as the generalized Wiener filtering algorithm but is faster because it does not require Fourier transform operations. In 2021, Manton et al.^137^ reported an equivalent AM signal demodulation method that used the structured illumination pattern as the carrier signal, the sample as the message signal, and the recorded data as the product of these two, i.e., the AM signal. They then heterodyned the AM signal with another sinusoidal pattern with the same phase and period as the carrier signal, realizing spectrum separation and recombination. Inspired by the series expansion of a function in mathematics^136^, Dan et al.^138^ proposed another SDR method by computing the coefficient matrix (Fig. 12, second row). Their results showed that this method reconstructed a superresolution image sevenfold faster than the FDR algorithm. However, this method did not address out-of-focus backgrounds. In 2022, Wang et al.^139^ developed a joint space and frequency reconstruction (JSFR)-SIM by combining spatial domain processing with optical sectioning superresolution SIM implemented in the frequency domain (Fig. 12, third row). By utilizing multithreading, they were able to reduce the execution time of reconstruction to 10.2 ms for raw images that were 512 × 512 pixels in size. Recently, the JSFR algorithm has been integrated with HiFi-SIM to form the joint space-frequency reconstruction-based artifact reduction algorithm for SR-SIM (JSFR-AR-SIM)^140^. However, all three SDR methods require parameter estimation, which is the most time-consuming step in the reconstruction process.

In contrast to these methods, Tu et al.^141^ developed a parameter-free algorithm called shifting phase SIM (SP-SIM), which directly reconstructs superresolution images in the spatial domain (Fig. 12, last raw). However, similar to the OS-SIM algorithm, the SP-SIM algorithm only preserves first-order spectral band information and discards the zero-order spectral band information during the derivation process. Consequently, low-frequency components of the reconstructed superresolution image using SP-SIM may be lost, resulting in lower image contrast compared to the FDR algorithm.

#### Open-source software

Open-source and open-access software packages for SR-SIM reconstruction have become more prevalent, and the once-opaque algorithms are now accessible to ordinary users. In this section, we provide a summary of existing 2D-SIM open-source reconstruction algorithms in Table 1, which includes information on the number of raw images needed, achievable resolution, implementation methods, and more.

**Table 1:** Open-source reconstruction algorithms for the 2D-SIM

Method	N/frames	Language	Resolution in X, Y/nm, or t/Hz	Property	Categories
**OpenSIM**^27^	9	MATLAB	~2-fold of the diffraction limit	Classical 2D-SIM	FDR
**OpenSIM-4**^126^	4	MATLAB	~2-fold of the diffraction limit	Frame reduction	FDR
**Hessian-SIM**^115^	9	MATLAB	A spatiotemporal resolution of 88 nm and 188 fps	Ultrafast and hour-long dynamic superresolution imaging	Iterative algorithm
**Sparse-SIM**^118^	9	MATLAB	~60 nm resolution at a frame rate of up to 564 fps	Sparse deconvolution	Iterative algorithm
**fairSIM**^89,90^	9/15-3D slice	Java	~2-fold of the diffraction limit	Classical 2D-SIM with notch filter	FDR
**VIGOR**^132^	9/15-3D slice	Java	Multicolor SR-SIM imaging at video frame- rates (25 reconstructed fps or more)	GPU-accelerated	FDR
**HexSIM**^131^	7	Python	Over 239 input raw images per second at 512 × 512 pixels, generating over 34 SR fps at 1024 × 1024 pixels	GPU-accelerated Frame reduction Hexagon illumination	SDR + FDR
**SP-SIM**^141^	9	MATLAB	~2-fold of the diffraction limit	No parameter estimation Suitable for speckle illumination pattern	SDR
**JSFR-SIM**^139^	9	MATLAB	The reconstruction time is 10.2 ms for raw images with 512 × 512 pixels	OS-SR-SIM	SDR + FDR
**SIM-Toolbox**^114^	9	MATLAB	~2-fold of the diffraction limit	classical OS-SIM 2D-SIM with homodyne detection	Iterative algorithm
**HiFi-SIM**^91^	9/15-3D slice	MATLAB	~2-fold of the diffraction limit	High fidelity PSF engineering	FDR
**MRA**^122^ **&DeepMRA**	9	MATLAB	~70 nm fidelity-ensured resolution	High fidelity Suppression of defocus background	FDR

Although some of the 2D-SIM reconstruction algorithms listed in Table 1 can reconstruct a single 3D image slice using a 2D-OTF, this does not constitute a true 3D-SIM image stack. This is because 3D-SIM reconstruction requires a system-specific 3D-OTF, preferably experimentally measured, to achieve high spatial resolution along the vertical axis. Without this information, the spatial resolution along the vertical direction may be limited.

In addition, several open-source ImageJ plugins offer tools for assessing the quality of SIM images. For example, SIMcheck can analyse both SIM raw and reconstructed data, providing system calibration to help users acquire optimum raw data for successful image reconstruction^142^. Another plugin, NanoJ-SQUIRREL (superresolution quantitative image rating and reporting of error locations), can quantify artifacts in SIM-reconstructed images^143^. By comparing a reference image (generally diffraction-limited) with a superresolution image, a quantitative map of localized image artifacts can be generated and used to guide researchers in optimizing imaging parameters.

Regarding resolution assessment methods, Koho et al.^144^ proposed a method based on Fourier ring correlation (FRC) analysis, where a single image is divided into four subsets (i.e., two-image pairs) and used to estimate the effective PSF in Wiener and iterative RL deconvolution. In 2019, Descloux et al.^145^ proposed a rapid image resolution estimation method called decorrelation analysis, which also uses a single image without prior knowledge. This method explores the highest frequency from the local maxima of the decorrelation functions, avoiding user-defined parameters. However, these methods may not be suitable for images with low SNR or artifacts, which could be interpreted as detailed information of the samples, leading to inaccurate estimated resolution.

**Fig. 13:**
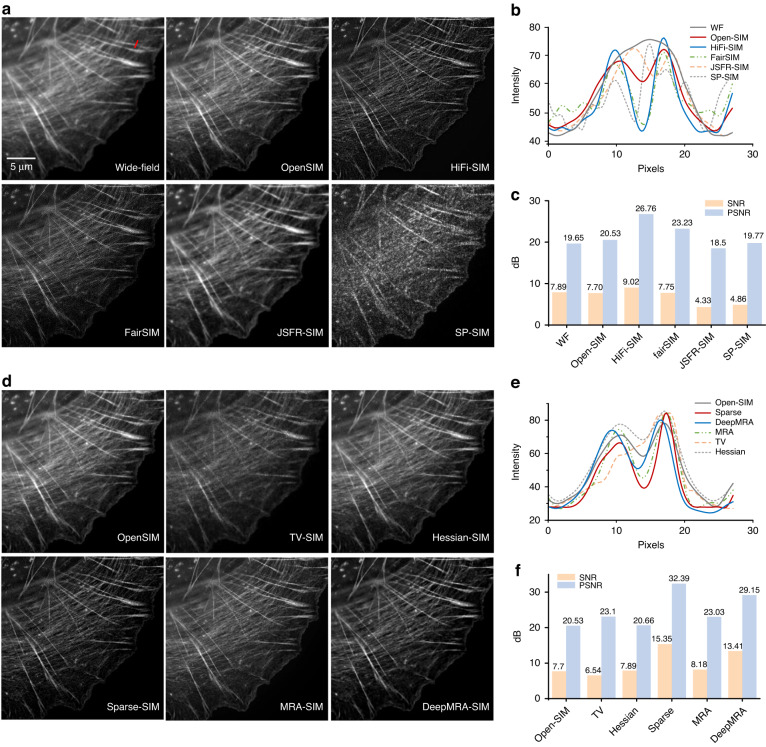
Reconstruction results of different 2D-SIM algorithms under high SNR conditions. **a** The results reconstructed under averaging, OpenSIM, HiFi-SIM, Fair-SIM, JSFR-SIM, and SP-SIM. **b** Intensity profiles along the red line in (**a**). **c** SNR and PSNR statistical diagrams of each result in (**a**). **d** The results obtained by postprocessing OpenSIM results using regularization-based iterative algorithms, such as TV, Hessian, sparse, MRA, and DeepMRA. **e** Intensity profiles along the red line in (**d**). **f** SNR and PSNR statistical diagrams of each result in (**d**)

**Fig. 14:**
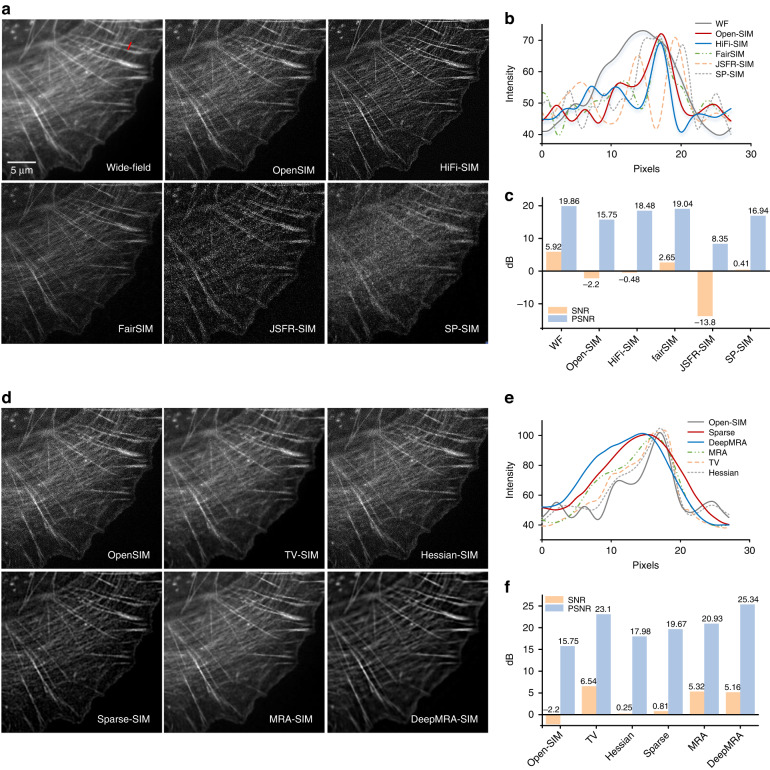
Reconstruction results of different 2D-SIM algorithms under low SNR conditions. **a** The results reconstructed under averaging, OpenSIM, HiFi-SIM, Fair-SIM, JSFR-SIM and SP-SIM. **b** Intensity profiles along the red line in (**a**). **c** SNR and PSNR statistical diagrams of each result in (**a**). **d** The results obtained by postprocessing OpenSIM results using regularization-based iterative algorithms, such as TV, Hessian, sparse, MRA, and DeepMRA. **e** Intensity profiles along the red line in (**d**). **f** SNR and PSNR statistical diagrams of each result in (**d**)

To analyse and compare the advantages and disadvantages of several open-source algorithms listed in Table 1, we processed two sets of raw data of actin filaments labelled with AF-568 phalloidin dye collected under high SNR (i.e., SNR = 7.7 dB, PSNR = 20.53 dB for OpenSIM) and low SNR (i.e., SNR = −2.2 dB, PSNR = 15.75 dB for OpenSIM). It is clear in Fig. 13a–c that in the case of a high SNR of the raw images, HiFi-SIM can better eliminate defocus information while maintaining high image resolution. Although the fairSIM algorithm has a faster reconstruction speed than HiFi-SIM and OpenSIM, the reconstructed image contains more artifacts. Regarding the SDR algorithm, JSFR-SIM provides a resolution improvement effect comparable to OpenSIM, but it cannot suppress defocus information as effectively as HiFi-SIM. SP-SIM has the fastest reconstruction speed but lacks zero-order information, causing a discontinuity in the reconstructed images, making it difficult to assess the resolution improvement effect. In the case of low SNR, as shown in Fig. 14a–c, noise introduced during the reconstruction process can reduce the resolution of the final reconstructed image. Overall, the FDR algorithm is more robust to noise than the SDR algorithm.

A regularization-based iterative algorithm is applied based on the reconstruction output of OpenSIM. In the case of a high raw image SNR (Fig. 13d–f), TV-SIM results in reduced resolution due to stepped artifacts, while Hessian-SIM maintains the resolution of the original OpenSIM output. Sparse-SIM, MRA, and DeepMRA can further enhance image resolution. In addition, Sparse-SIM and DeepMRA can effectively suppress defocus information. When the raw image SNR is low (Fig. 14d–f), denoising reduces the resolution of the final image, regardless of the algorithm used. There is a trade-off between noise suppression and contrast enhancement. We organize the evaluation results of the above various methods in Appendix 3.

### Development of 3D-SIM reconstruction methods

Recently, the problem of multilayer 3D-SIM image reconstruction has been addressed and implemented. For example, in 2015, based on the generalized Wiener reconstruction theory mentioned in ref. ^12^, Shao et al. developed and shared the 3D-SIM reconstruction software with CUDA acceleration^133^. This method has been widely applied in biological study^146,147^. In 2021, Smith et al.^148^ presented a physically realistic noise model and provided three complementary reconstruction methods: true-Wiener-filtered SIM, flat-noise SIM, and notch-filtered SIM. Experimental results demonstrated that introducing notch filtering can partly overcome the trade-off between increasing contrast and suppressing noise. In the same year, Zhu et al. proposed an iterative algorithm called NGD-SIM based on gradient descent and a nonlinear optimizer RMSprop^149^. However, this algorithm is time-consuming. In 2022, Cai et al. proposed a TV-FISTA-SIM algorithm that combines TV with the fast iterative shrinkage threshold algorithm (FISTA)^150^ to further improve imaging speed. Compared to the NGD-SIM algorithm, this algorithm achieves faster convergence speed and higher reconstruction fidelity when the SNR is as low as 5 dB. Additionally, in 2022, Cao et al.^151^ proposed an Open-3DSIM algorithm by introducing spectrum filtering to further optimize the reconstruction results of traditional 3D-SIM and improve its friendliness to general users. They provided a MATLAB code, ImageJ version, and Exe application simultaneously. Experimental results demonstrated that Open-3DSIM has superior performance in suppressing artifacts and removing defocus information.

It is important to consider the effect of motion artifacts on the quality of a reconstructed image if the sample is moving during imaging^54^. In wide-field microscopy, small sample movements may go unnoticed if they are smaller than the resolution limit. However, in SIM reconstruction, even low velocities may introduce artifacts, leading to a reduction in resolution and potentially misleading interpretations. Thus, Förster et al. proposed a frame difference method (FDM) ^152^ and its improved versions^153^ to detect and locate motion artifacts in SIM images. However, these methods require 3D-stack data and are not executable for two-beam methods, such as nonlinear SIM. Furthermore, to reduce artifacts resulting from optical aberrations and enable 3D-SIM imaging in thick tissues, Lin et al.^105^ proposed the AO-3DSIM system, which combines adaptive optics with 3D-SIM and processes 3D-stack data using the generalized Wiener filtering method. The AO-3DSIM system achieved a resolution of 150 nm laterally and 570 nm axially, along with optical sectioning, at a depth of 80 μm through *Caenorhabditis elegans*, compared to a resolution of 280 nm laterally and 930 nm axially in wide-field imaging. Table 2 summarizes the corresponding 3D-SIM open-source reconstruction methods for 3D-SIM.

**Table 2:** Open-source reconstruction algorithms for the 3D-SIM

**Method**	**Categories**	**Language**	**Property**
3D-SIM^133^	FDR	CUDA C++ acceleration	Classical 3D-SIM
AO-3DSIM^105^		Python	Classical 3D-SIM
Open-3DSIM^151^		Fiji/MATLAB	Suppression of noise artifacts Spectrum optimization
True-Wiener-filtered SIM^148^		MATLAB	High contrast imaging
Flat-noise SIM^148^			Suppression of structural noise artifacts
Notch filtered SIM^148^			Higher image contrast than flat-noise SIM
TV-FISTA-SIM^150^	Iterative algorithm		Fast convergence speed

**Fig. 15:**
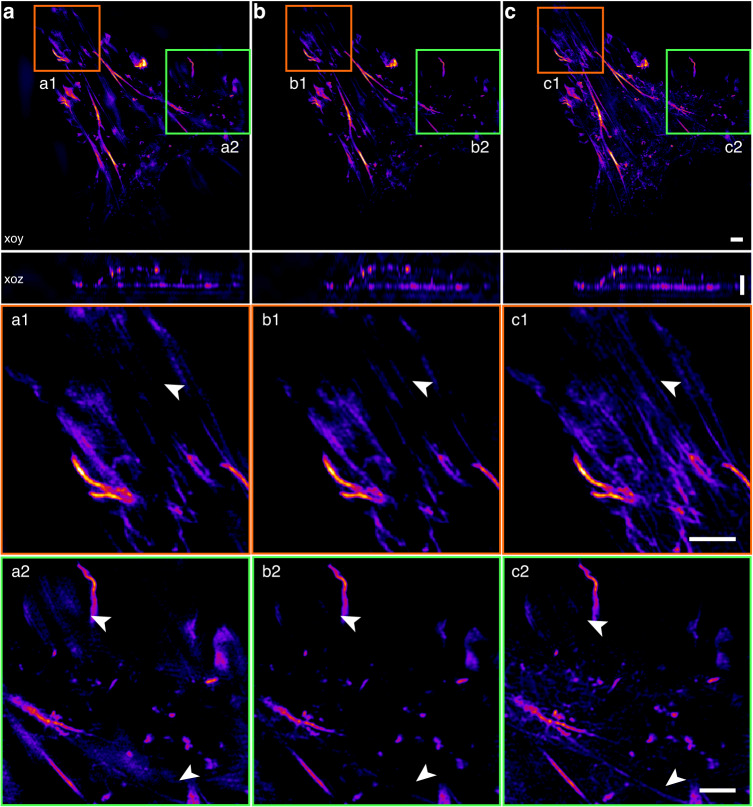
Reconstruction results under three typical 3D-SIM algorithms. **a** AO-3DSIM, **b** True-Wiener-filtered SIM, and **c** Open-3DSIM. **a**1–**c**1 Magnified images from the orange box in (**a**–**c**); **a**2–**c**2 Magnified images from the green box shown in (**a**–**c**)

We compared and analysed three open-source 3D-SIM reconstruction algorithms, AO-3DSIM, SIMnoise (i.e., true-Wiener-filtered SIM), and Open-3DSIM, by testing another actin filament sample obtained from the open-source data of ref. ^105^. As shown in Fig. 15, the image reconstructed under the AO-3DSIM algorithm contained some artifacts caused by out-of-focus information (Fig. 15a). Although these artifacts can be effectively removed by using SIMnoise, some details of the sample are also lost (Fig. 15b). Open-3DSIM can better retain the detailed information of the sample while suppressing the defocus information (Fig. 15c). However, it should be noted that the comparison results may vary depending on the specific sample and imaging conditions. Therefore, users should choose the appropriate algorithm based on their own requirements and considerations, such as speed, accuracy, noise suppression, and artifact reduction. Moreover, further development and optimization of 3D-SIM reconstruction algorithms are still needed to achieve higher resolution, faster computation, and higher robustness.

### Development of blind-SIM reconstruction methods

If the raw image SNR is too low, or the illumination patterns are distorted due to the inhomogeneity of the sample refraction index, parameter estimation-based reconstruction algorithms may fail to work. To overcome this problem, Mudry et al.^154^ developed a blind-SIM reconstruction method in 2012 for illuminating samples with random light speckles. This method dramatically simplifies the experimental setup by not requiring knowledge of the illumination pattern. However, the temporal average of speckle illumination must be roughly homogeneous over the sample for it to work, limiting its wide application. In 2013, Min et al.^155^ presented another speckle illumination microscopy by implementing a multiple sparse Bayesian learning (M-SBL) algorithm^156^. A threefold resolution gain was reported under the joint support constraints. Moreover, Ayuk et al.^157^ extended the application of blind-SIM to periodic illumination patterns by introducing an additional Gaussian filter during the inversion procedure. It was shown that this filtered blind-SIM is as efficient as traditional SIM when the illumination pattern is periodic. Additionally, it is robust to distortion and misalignment.

In 2014, by improving the Fourier ptychography (FP) algorithm proposed by Zheng et al.^158^, Dong et al.^159^ proposed a pattern-illumination Fourier ptychography (piFP) method. This method is applicable to any unknown illumination pattern and has been used in computational photography and image-based rendering^160^. In 2015, Ströhl et al.^161^ presented a jRL-MSIM plan to suppress out-of-focus signals. Later, Chakrova et al.^162^ compared the piFP and jRL algorithms by formulating a generalized maximum likelihood estimation (MLE). They found that the piFP method can resolve periodic and isolated structures equally well, while the jRL method is more suitable for processing isolated objects.

In a similar fashion to the piFP method, subsequent methods such as PE-SIMS^163^ (a self-calibration strategy for SIM) and TIRF-piFPM^164^ were proposed. However, these methods require prior knowledge of the illumination pattern. In 2021, Samanta et al.^165^ envisaged the utility of optical lattice illumination patterns generated by phase-engineered interference of coplanar beams^166^ and presented a blind reconstruction approach combined with a multiple signal classification algorithm (MUSICAL)^167^. The results demonstrated that using sinusoidal and multiperiodic illumination patterns, a maximum of three- and six-fold resolution enhancement beyond the diffraction limit could be obtained, respectively.

While out-of-focus signals can be addressed by introducing OTF attenuation^89^ or RL deconvolution^161,162^, they are only suitable for imaging relatively thin samples. To address out-of-focus in thicker samples, several blind-SIM algorithms have been proposed^168–171^. For example, Jost et al.^168^ proposed the thick slice blind-SIM algorithm, which considers several additional planes to collect out-of-focus light and processes monofocal layer data to bridge 2D- and 3D-SIM reconstructions. In 2019, Soubies et al.^171^ improved upon this method by proposing an inner-loop-free alternating-direction method of multipliers (ADMM)^172^, which relies on a specific formulation of the optimization problem and closed-form expressions of proximal operators, resulting in faster computation. By considering additional planes in the model, they demonstrated improved image quality for slice-by-slice computational sectioning.

## The combination of SIM with other techniques

Although SIM technology provides unprecedented access to the inner world of cells and various biological processes, it relies on relatively sophisticated optical setups and rigorous experimental conditions. Other superresolution imaging modalities that can be combined with SIM have been proposed to reduce the cost for research labs and further improve the quality of SIM reconstructions, as well as increase imaging speed, depth, and resolution.

### Combination with other SR optical systems

In addition to TIRF, surface plasmons, STED, and other techniques, combining SIM with two-photon excitation enables deeper imaging depth and better contrast compared to single-photon excitation in thick scattering samples, such as Drosophila melanogaster larval salivary glands and mouse liver tissue^173–175^. For example, two-photon instant SIM (2P-ISIM)^175^ can provide a spatial resolution of ~150 nm laterally and ~400 nm axially and a frame rate of ~1 Hz at depths exceeding 100 μm from the coverslip surface in thick samples. In addition, in 2017, Gregor et al.^173^ improved the frame rate of 2P-SIM to 30 Hz by incorporating nonlinear excitation and a single resonant scanner. However, the high peak intensity in 2 P excitation results in increased phototoxicity, limiting the long-duration imaging of 2P-SIM. Additionally, 2P-SIM is challenging to use for multicolor imaging owing to the spectral matching limitation between laser sources and fluorescent probes^176^.

In 2017, Chang et al.^177^ introduced SIM to light-sheet-based fluorescence microscopy (LSFM) and achieved sub-100 nm lateral resolution while significantly enhancing axial resolution. The lateral resolution can be further enhanced by combining 2D-SIM with intensity correlation microscopy (ICM)^178^ or SOFI^179^. Classen et al. also demonstrated that by connecting with ICM^180^, the axial resolution of a 3D-SIM system was improved. In 2018, Wang et al.^181^ proposed the ExM-SIM method, which combines expansion microscopy with SIM. They also presented protocol details and steps to analyse protein localization using ExM-SIM and analysed the 3D organization of multiprotein complexes with ~30 nm lateral resolution. In 2020, Helle et al.^182^ proposed a chip-based SIM (cSIM) method using a photonic integrated circuit (PIC) chip to create standing wave interference patterns. As the cSIM frequency shift is governed by the interference angle and the refractive index of the waveguide material itself, it can further extend the resolution provided by conventional SIM.

In 2021, Pilger et al.^183^ developed striped-illumination patterns in two-photon laser scanning microscopy (2P-LSM) using an sCMOS camera and a customized scanning protocol. This technique can be exploited to achieve optical superresolution and contrast enhancement. In 2022, Wang et al.^184^ proposed a multiphoton SIM (mP-SIM) method utilizing a nonsinusoidal structured illumination pattern and associated reconstruction algorithm. Their results on nanoparticles and bovine pulmonary artery endothelial (BPAE) cells with stained F-actin demonstrated an 86 nm lateral resolution for 2P-SIM and a 72 nm lateral resolution for second-harmonic-generation (SHG)-SIM.

### Combination with deep learning techniques

SR-SIM reconstruction typically requires extensive computational postprocessing of acquired image data and a physical model of the image-formation process. As a result, it is usually time-consuming with high computational expenses. While more accurate models yield higher-quality results, there is often a trade-off between the level of accuracy and the exhaustive parameter search and computational cost. The advent of deep learning, particularly deep convolutional neural networks (CNNs), has provided new solutions for image analysis. In microscopy, CNNs can tackle the pseudoinverse imaging problem of image transformation processes. Moreover, they can learn the stochastic characteristics of optimal solutions by leveraging paired end-to-end transformation images^185,186^. Recently, researchers have explored the potential of CNNs to augment SIM in terms of speed and low SNR.

In 2019, Wang et al.^187^ presented a deep learning-based framework to achieve superresolution and cross-modality image transformations in fluorescence microscopy by training a generative adversarial network (GAN) model. This framework can learn pixel-to-pixel transformations and enhance resolution while avoiding potential artifacts by incorporating a highly accurate multistage image registration and alignment process. However, GANs are generally challenging to train because they require delicate balancing of a generator (G) and discriminator (D), and more input images and training epochs than conventional CNNs. In 2020, Jin et al.^188^ proposed U-Net-based frameworks, namely, U-Net-SIM3 and U-Net-SIM15, which reduced the number of raw images by 5-fold and retrieved superresolution information from low-light samples. Compared to other CNNs, U-Net-based frameworks are more user-friendly for biologists and users with less deep learning experience.

In 2021, Qiao et al.^189^ developed a deep Fourier channel attention network (DFCAN) and its derivative DFGAN, which were trained using a GAN strategy. Unlike other methods that use structural differences in the spatial domain, DFCAN and DFGAN leverage frequency content differences across distinct features in the Fourier domain to learn hierarchical representations of high-frequency information. The experimental results demonstrated that DFCAN and DFGAN can infer superresolution images of diverse biological structures more precisely than U-Net-based frameworks. In addition, they can reconstruct high-quality superresolution live-cell images that capture the dynamic interactions between intracellular organelles and the cytoskeleton over a tenfold longer duration relative to conventional SIM. The authors suggest that non-GAN models (i.e., DFCAN) are more appropriate for low- to medium-fluorescence imaging conditions, generating superresolution images with good quantifiability. In contrast, a GAN model (i.e., DFGAN) may be preferable for specimens with high structural complexity, provided it offers comparable results to conventional SIM. However, the disadvantage is that multiple Fourier transform operations demand significant computing resources and time, especially when applied to 3D-SIM data.

Shah et al.^190^ presented two robust end-to-end deep-learning workflows, SR-REDSIM and RED-fairSIM, utilizing a residual encoding-decoding convolutional neural network (RED-Net). These networks were robust against different noise intensities without needing preprocessing image procedures. However, the final output denoised raw image barely exhibits Moiré fringes, leading to a failure to reconstruct superresolution information in SIM images. To address this, several approaches have been proposed to improve the robustness of SIM reconstruction to noise and illumination pattern irregularities. These include a transfer learning-based generality end-to-end deep residual neural network ML-SIM^191^, a custom convolutional neural network architecture BS-CNN for blind-SIM reconstruction^192^, and a modified residual channel attention network (RCAN)^193^ by Boland et al.^194^. The RCAN was able to reconstruct 3D-SIM image stacks with double the axial resolution of existing 2D-SIM reconstructions without compromising lateral resolution or structural fidelity.

To improve imaging speed by reducing the number of raw images, several methods have been proposed. One approach is to use the cycle-consistent generative adversarial network (cycleGAN)^195^, which can reconstruct a superresolution image through the single-direction phase shift of only three raw SIM frames instead of the traditional nine. Another method is the channel attention generative adversarial network (caGAN) based on the spatial channel attention mechanism^196^, which can achieve a comparable or higher quality of 3D-SIM reconstruction under low SNR and high out-of-focus background conditions while using axially downsampled raw images compared to the conventional algorithm. Single-shot SIM reconstruction methods based on multiple networks have also been proposed, such as the combination of a GAN and DU-Net^197^, where the GAN generates other structured illumination images from a single raw image, and DU-Net reconstructs superresolution images from these generated images. Additionally, a fast and lightweight SIM superresolution network (FLSN) has been developed^198^, including a noise estimation subnetwork and Haar wavelet-based bandpass attention modules. The experimental results demonstrated that SF-SIM is almost 14 times faster than traditional SIM methods while achieving similar results.

Note that image transformation in deep learning superresolution models is an ill-posed problem. Although these models leverage a large amount of well-registered data to learn good statistical transformation, it is theoretically impossible for network inference to obtain ground truth images in every detail. This poses a great challenge in replacing superresolution microscopy entirely with computational-only approaches^189^. In 2022, Qiao et al.^199^ developed rationalized deep learning (rDL) for SIM by incorporating prior knowledge of illumination patterns into network training and inference, reducing the ill-posedness of the final superresolution image. Compared to GAN-based models such as DFGAN, rDL reduced model uncertainty by fivefold. The experimental results demonstrated that rDL SIM could eliminate spectral bias effects and improve the resolution of reconstructed superresolution images. Moreover, rDL SIM enhanced the modulation depth of illumination patterns and was robust to unexpected variations, such as initial phase error and spherical aberrations, when compared to conventional SIM reconstruction algorithms such as Hessian-SIM. According to the functions and characteristics of the networks, we have summarized the aforementioned methods, as presented in Table 3.

**Table 3:** SIM combined with deep-learning techniques

Framework	Functionality	Features
**GAN**^187^	Superresolution and cross-modality image transformations	It learns a pixel-to-pixel transformation and resolution enhancement while avoiding potential artifacts.
**U-Net-SIM3**^188^	Speed up SIM reconstruction	Fivefold reduction in the number of raw images
**U-Net-SIM15**^188^	Robust to noise	Superresolution information is retrieved from the low-light sample
**DFCAN & DFGAN**^189^	Infer superresolution images of diverse biological structures more precisely than the U-Net network	It captures dynamic interactions between intracellular organelles over a tenfold longer duration relative to conventional SIM
**SR-RED-SIM**^190^	Robust to noise Low training costs	It is robust against different noise intensities without needing a preprocessing image procedure
**RED-fairSIM**^190^	A combination of fairSIM and RED-Net	Any retraining or fine-tuning is not needed, even if the SNR differs between training and application.
**ML-SIM**^191^	Robust to noise and irregularities in the illumination patterns	SIM reconstruction is based on transfer learning without fine-tuning or necessary retraining
**BS-CNN**^192^	blind-SIM reconstruction	It outperforms other deconvolution algorithms and is robust in cross-database variability
**Modified RCAN**^194^	3D-SIM reconstruction	Axial resolution doubles that of current 2D-SIM reconstructions without loss of lateral resolution
**cycleGAN**^195^	Speed up SIM reconstruction	It has higher training efficiency than U-Net-SIM3
**caGAN**^196^	Robust to noise Requires fewer computing resources	It can reconstruct high-quality 3D-SIM images using the axially downsampled raw images under low SNR and high out-of-focus background.
**GANs and DU-Net**^197^	Single-frame SIM reconstruction	GANs were trained to generate other structured illumination images from a single raw image, and DU-Net was trained to reconstruct superresolution image
**FLSN**^198^	Single-frame SIM reconstruction	SIM reconstruction using extreme low-light and short-exposure frames is 14 times faster than traditional SIM methods when achieving similar results
**rDL**^199^	Robust to noise, model uncertainty, and estimation error of illumination pattern parameters	It can be applied to rationally denoise the raw images, mainly to reduce the ill-posedness of the final superresolution image

## Summary

### Comparison of off-the-shelf SIM systems

**Fig. 16:**
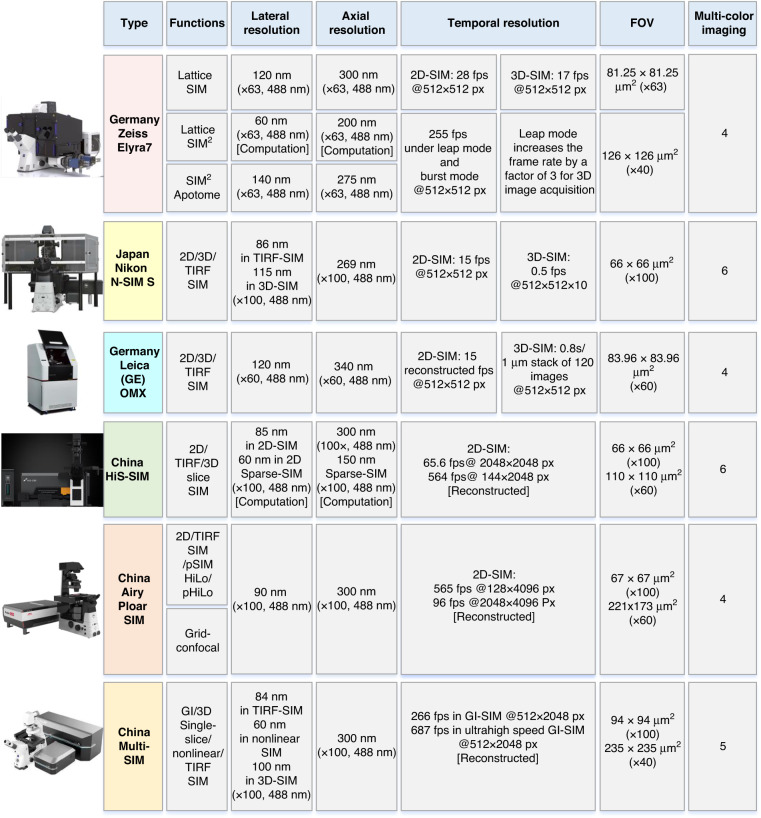
Comparison of representative commercial SIM systems. Compare the following parameters of the system: resolution, imaging speed, imaging FOV and multi-color imaging capability. The term [computation] refers to the resolution achieved through postprocessing algorithms applied to the traditional reconstruction results

We have benchmarked the state-of-the-art performance of a typically accessible technique using commercially available systems. In addition, we have summarized and compared several representative off-the-shelf SIM systems based on their resolution, imaging speed, imaging FOV, and multicolor imaging capabilities^200–205^, as shown in Fig. 16.

Among these systems, Elyra 7 stands out for its faster imaging speed and lower phototoxicity owing to the use of a lattice illumination pattern^205^. The reconstruction resolution is further improved in the ‘HiS-SIM’ system, which combines a sparse deconvolution algorithm^203^. It is worth noting that the ‘Airy Polar-SIM’ system^200^ introduces polarization imaging^206^, while the “Multi-SIM” system^204^ offers nonlinear imaging, both of which demonstrate distinct features for resolving subcellular organelle structures. The diversified development of unique SIM systems also offers a promising avenue for addressing critical problems that cannot be answered using conventional techniques.

### Conclusion and perspective

SIM, a revolutionary concept borrowed from electric signal processing to optical super-resolution microscopy, has emerged as a novel imaging technology with remarkable capabilities in terms of field-of-view, speed, and compatibility with fluorescent dyes. In this article, we present an overview of two SIM algorithms, namely, OS-SIM and SR-SIM, and their implementation modalities using linear SIM as an example. We also briefly review existing OS-SIM processing algorithms before delving into the development of SR-SIM reconstruction algorithms. While it is nearly impossible to cover all algorithms, we have included representative methods for each SR-SIM reconstruction category in each domain. The methods are categorized into 2D-SIM, 3D-SIM, and blind-SIM. Regarding 2D-SIM, we subdivide it into three parts: parameter estimation, FDR (generalized Wiener filtering, regularization-based iterative optimization methods), and SDR. Finally, we summarize various combinations of SIM with other techniques to optimize the imaging strategy for better spatial/temporal resolution, deeper image depth, and faster imaging speed.

When imaging thin biological samples (<10 μm), SR-SIM algorithms with careful postprocessing provide superior spatial resolution. On the other hand, OS-SIM algorithms are suitable for suppressing out-of-focus information in thick biological samples (~10–~100 μm). However, for thicker (>100 μm) biological samples or those with higher levels of scattering, it may be necessary to use multiphoton illumination and adaptive optical correction. Given the vast number of SR-SIM reconstruction algorithms available—both open-source and commercial—choosing the “right algorithm for processing data from a sample” can be a challenging task. Nevertheless, after surveying the numerous techniques, some general trends have become clear (Figs. 13–15).

For fixed biological samples with high SNR, e.g., probing the fine structure of the actin cytoskeleton in the cell, HiFi-SIM outperforms other algorithms based on Wiener filtering. It can effectively remove out-of-focus information, improve the resolution of the reconstructed image, and retain sample details. However, it should be noted that a single 3D image slice reconstructed using HiFi-SIM is not a true 3D-SIM image. To achieve simultaneous improvement in axial and lateral resolution, multilayer 3D-SIM reconstruction algorithms are needed, such as Open-3DSIM and AO-3DSIM. However, these methods require a more complex hardware setup and more raw images (taken at several focal planes) for reconstruction. Hence, new SIM systems and innovative reconstruction algorithms are needed to simplify the experimental constraints of 3D-SIM and speed up the reconstruction process. Additionally, NL-SIM can be utilized to further enhance the resolution of the reconstructed image. However, saturated SIM requires the extremely high light intensity to accelerate photobleaching, and photoswitchable fluorophores can make sample preparation more cumbersome in an application. Therefore, developing novel fluorescent dyes that can tolerate many on-off cycles or exploring SAN-SIM without specific fluorescent dyes, as well as enhancing STED-SIM with low depletion laser power, could be promising areas of future research.

Faster imaging speed implies shorter signal accumulation time and lower SNR in acquired images. To improve the observation of the high-speed movement of samples such as mitochondria and endoplasmic reticulum, as well as the dynamics of interaction between mitochondria and the actin cytoskeleton in cells, it may be beneficial to use regularization-based iterative optimization methods. Techniques that incorporate rolling reconstruction, such as Hessian-SIM, Sparse-SIM, MRA, and DeepMRA, are worth considering. However, these methods rely on ad hoc tuning of parameters for different samples, which can be challenging for beginners. In addition, when the reconstructed image has relatively low SNR (i.e., SNR = −2.2 dB), their spatial resolution is often compromised for improved SNR. A physically realistic noise model that can explain noise propagation through SIM reconstruction and compensate for image noise is needed to improve the approach. Furthermore, although an SDR algorithm can incorporate GPU acceleration for dynamic measurements, its reconstruction quality is not as good as FDR results. Exploring the potential of SDR algorithms and developing novel algorithms could bridge the gap and enhance imaging speed.

If a biological sample is tested under complicated experimental scenarios, such as illumination pattern drift or constant adjustment of the region of interest and focus, GPU-accelerated reconstruction methods that estimate illumination parameters in advance and reuse them in subsequent reconstruction may no longer be applicable. In contrast, PCA-SIM, as a noniterative, fast parameter estimation method, can run and update the illumination parameter estimation in real-time. However, if the illumination patterns are distorted due to the inhomogeneity of the sample’s refractive index, blind-SIM reconstruction methods should be considered. A drawback of these methods is that they are typically slower than the regularization-based iterative optimization methods due to the need for complex and computationally expensive deconvolution algorithms to ensure the convergence of the iterative algorithm. Thus, it is necessary to explore more simplified blind-SIM reconstruction algorithms for dynamic live-cell measurements.

Recently, neural network-based deep-learning models have been developed and demonstrated to augment SR-SIM in terms of speed and low SNR. Although some papers and researches have demonstrated the outstanding performance of learning-based superresolution microscopy in various image transformation tasks, such as denoising and image superresolution^187,207^, these tasks are essentially ill-posed problems^186^, indicating that several solutions exist for a given input in the high-dimensional manifold of all possible inferences. Enough datasets for generalized network performance is thus needed and the acquisition of high-quality ground truth (GT) for training DNNs is not trivial in most bioimaging applications. Moreover, it is still unclear to what extent the information conveyed by deep learning superresolution images can be leveraged for quantitative analysis, and under what conditions these approaches are superior to conventional superresolution microscopy. Recent studies on rDL methods have demonstrated that combining physical models can decrease uncertainty and yield physically feasible inferences. As a result, we anticipate further improvements in rDL techniques to bridge the gap between deep learning-based and physically realistic models.

## Appendix 1

**Cell Maintenance and Preparation**

U2OS and COS7 cells were cultured in complete cell culture medium composed of high glucose medium DMEM (Gibco, 11995-040) supplemented with 10% foetal bovine serum (Gibco, 10099) and 1% penicillin-streptomycin antibiotics (10,000 U/mL, Gibco, 15140148). The cells were incubated at 37 °C and 5% CO_2_ until they reached 75% confluency. For fixed-cell imaging experiments, cells were seeded onto coverslips (Thorlabs, CG15CH2). For live-cell imaging experiments, cells were seeded in μ-Slide 8 Well (Ibidi, 80827).

**Labeling Actin in Fixed U2OS Cells**

U2OS cells were fixed with 4% formaldehyde (Invitrogen, R37814) for 15 min at room temperature when they reached 75% confluency. After that, the cells were permeabilized with 0.1% Triton™ X-100 (Invitrogen, HFH10) in PBS for 5 min, rinsed with PBS, and then stained with Alexa Fluor 568 Phalloidin (Invitrogen, A12380)/Alexa Fluor 488 Phalloidin (Invitrogen, A12379) dyes to label the actin filaments for 1 h at room temperature. The samples were washed twice with PBS to remove excess dye, and the coverslips were air-dried in the dark. Finally, 30 µL of Prolong (Invitrogen, P36984) mounting medium was added to the coverslips and left to air-dry overnight at 4 °C before observation.

## Appendix 2

**Table:**

Abbreviation	Full description
SIM	structured illumination microscopy
OS-SIM	optical sectioning SIM
SR-SIM	superresolution SIM
SMLM	single-molecule localization microscopy
STED	stimulated emission depletion microscopy
TIRF	total internal reflection fluorescent
HiLo	hybrid illumination
fps	frames per second
2D-SIM	two-dimension/two-beam SR-SIM
FDR	Fourier domain reconstruction algorithm
SDR	spatial domain reconstruction algorithm
HiFi-SIM	high-fidelity SIM
TV	total variation
MAP	maximum a posteriori
sorSIM	second-order optimally regularized SIM
Bi-CGSTAB	Biconjugate gradient descent algorithm
MLE	maximum likelihood estimation
SIMILR	SIM with an interleaved reconstruction strategy
JSFR-SIM	joint space and frequency reconstruction SIM
SP-SIM	shifting phase SIM
3D-SIM	three- dimension/three-beam SR-SIM
NL-SIM	nonlinear SIM
RMS	root mean square
SNR	signal-to-noise ratio
DSI	dynamic speckle illumination
picoSIM	polarization-illumination-coded SIM
LiMo	line-illumination modulation microscopy
fMOST	fluorescent micro-optical sectioning tomography
DMD	digital micromirror device
MF SIM	moving fringe SIM
SLM	spatial light modulators
iSIM	instant SIM
EOM	electro-optics modulator
PA NL-SIM	patterned activation of photoswitchable fluorophores NL-SIM
PSIM	plasmonic SIM
SAN-SIM	saturable absorption-assisted nonlinear SIM
STED	stimulated emission depletion
SPR	surface plasmon resonance
SSTED-SIM	structured-excitation STED-SIM
RL	Richardson–Lucy
CNN	convolutional neural network
COR	cross-correlation
POP	phase of peak
ACR	autocorrelation
IRT	image recombination transform
PCA	principal component analysis
TV	total variation
MAP	maximum a posteriori probability
FRC	Fourier ring correlation
FISTA	fast iterative shrinkage threshold algorithm
FP	Fourier ptychography
ADMM	alternating-direction method of multipliers
ICM	intensity correlation microscopy
GAN	generative adversarial network

## Appendix 3: Evaluation of reconstructed results

**Table:**

Method		High SNR	Low SNR
FDR-generalized Wiener filtering	HiFi-SIM	No defocus information while maintaining sample detail	The quality and resolution of the reconstructed images are degraded by noise
	Open-SIM	Cannot suppress defocus information	
	fairSIM	Can suppress defocus information, but has more artifacts	
SDR	JSFR-SIM	Can achieve the same resolution as OpenSIM, but the ability to suppress defocus information is not as good as HiFi-SIM	More susceptible to noise, reconstruction results are almost completely submerged in noise
	SP-SIM	Reconstruction results are low contrast and discontinuous	
FDR- regularization-based iterative algorithm	TV-SIM	Reduced resolution of reconstructed image due to stair-step artifacts	Reduced resolution of reconstructed image due to stair-step artifacts
	Hessian-SIM	Maintains the resolution of the original OpenSIM output	The quality of the reconstructed images is degraded by noise
	Sparse-SIM	Can further enhance the image resolution and effectively suppress defocus information	There is a trade-off between noise suppression and contrast enhancement
	MRA	Can further enhance the image resolution	
	DeepMRA	Can further enhance the image resolution and effectively suppress defocus information	
